# Cell Wall Proteome of *Candida albicans* Reveals Proteins Associated with Tolerance to Antibiofilm Activity of a *Lippia graveolens* Kunth Stem Extract

**DOI:** 10.3390/pathogens15020216

**Published:** 2026-02-14

**Authors:** Alejandra García-Núñez, Ana Lilia Martínez-Rocha, Carlos Antonio Alba-Fierro, Francisco Javier Ríos-Fránquez, Juan Pablo Cabral-Miramontes, María Estela Frías-Zepeda, Miguel Ángel Escobedo-Bretado, Estela Ruiz-Baca

**Affiliations:** 1 Facultad de Ciencias Químicas, Universidad Juárez del Estado de Durango, Av. Veterinaria S/N, Durango 34120, Mexico; alejandra.garcia.nunez@ujed.mx (A.G.-N.); carlos.alba@ujed.mx (C.A.A.-F.); javier.rios@ujed.mx (F.J.R.-F.); juan.cabral@ujed.mx (J.P.C.-M.); estela.frias@ujed.mx (M.E.F.-Z.); miguel.escobedo@ujed.mx (M.Á.E.-B.); 2 Departamento de Biología, División de Ciencias Naturales y Exactas, Universidad de Guanajuato, Guanajuato 36050, Mexico; anamartinez@ugto.mx

**Keywords:** *Candida albicans*, biofilms, cell wall proteins, *Lippia graveolens*, antifungals

## Abstract

*Candida albicans* is an opportunistic fungus capable of forming biofilms that are resistant to conventional antifungal treatments. This study evaluated the antibiofilm activity of an ethanolic extract from Mexican oregano (*Lippia graveolens* Kunth) stem and its impact on the protein composition of the *C. albicans* cell wall (CW). The proteomic analysis was restricted to the adherent cells that persisted after treatment, representing the more tolerant subpopulation. First, the biofilm-forming capacity of 18 clinical isolates was characterized. Subsequently, the effect of the *Lippia graveolens* Kunth stem extract on biofilm formation was assessed in clinical isolates of *C. albicans* with high and moderate biofilm-forming capacities. The results showed an MIC_90_ of 16 mg/mL against *C. albicans* isolates in planktonic growth. Furthermore, the extract exhibited an antibiofilm effect, showing a 77% inhibition in the highly biofilm-forming *C. albicans* 2400H strain at 1.6 mg/mL. To assess how the extract influences CW composition, we analyzed the CW proteome of the adherent biofilm cells of *C. albicans* that persisted after exposure to the stem extract. Mass spectrometry identified 1006 total proteins, where 156 were up-regulated, and 94 were down-regulated. Of the differentially expressed proteins, we identify 40 CW proteins (CWP’s) associated with dimorphic transition, adhesion, oxidative stress, and biofilm formation. These findings provide the potential of the *L. graveolens* Kunth stem as a natural antifungal agent against *C. albicans* biofilms. In addition, we identified CWP’s associated with tolerance to the extract’s antibiofilm activity, offering mechanistic insight into persistence and potential targets for improving antibiofilm interventions.

## 1. Introduction

*Candida albicans* is a dimorphic pathogenic fungus commonly present on the surface of organs such as the mouth, skin, upper respiratory tract, intestines, vagina, etc. Candidiasis is considered the most common among fungal infections, mainly in hosts with an immunological disorder [1]. One of the most important virulence factors of *C. albicans* is its ability to produce biofilms, which confer resistance to hostile environments when exposed to the host’s immune response or during treatment with antifungal agents [2,3]. The biofilm is characterized by a compact community of different cell types, such as yeast, pseudohyphae, and hyphae, enclosed in an extracellular polymeric matrix, preventing or reducing drug diffusion [4]. As a worrying fact, most of the drugs used today are ineffective against *C. albicans* biofilms [5]; therefore, searching for new bioactive molecules with therapeutic applications against this fungus has become a relevant issue.

Plant extracts and their compounds are a valuable source of products with therapeutic potential, which can be used to treat diseases such as candidiasis [6]. The biologically active compounds include primary and secondary metabolites such as acetogenins, alkaloids, flavonoids, coumarins, lactones, anthraquinones, glycosides, tannins, and phytosterols [7,8]. The inhibitory effect on the growth and biofilm formation of *C. albicans* has already been demonstrated using oil extracts from plants from the *Lippia* genus such as *Lippia junelliana*, *Lippia alba*, *Lippia salviaefolia*, *Lippia velutina*, *Lippia balansae*, *Lippia lasiocalycina*, *Lippia lupulina*, *Lippia citriodora*, *Lippia origanoides*, etc. [9,10,11,12,13,14,15]. Oregano is an aromatic plant whose main species include *Origanum vulgare*, *Lippia berlandieri* Shauer, and *L. graveolens* Kunth, the latter commonly referred to as Mexican oregano [16]. The leaves of *L. graveolens* Kunth are widely used as a condiment in fresh and processed foods, as well as in the production of essential oils [17]. Secondary metabolites such as carvacrol and thymol, which are obtained from the essential oil, contain high levels of antimicrobial activity, with thymol being the most active [18]. However, although oregano essential oils obtained from leaves have shown their effectiveness in reducing the growth of *C. albicans* and its biofilm formation, they can show undesirable effects, such as cytotoxicity in human cell lines [19]. The stem of *L. graveolens* Kunth plant, a by-product of essential oil extraction, has no commercial value despite being rich in compounds such as caffeic acid and 2-hydroxybenzoic acid and flavonoids such as naringenin, taxifolin, eriodyctiol, acacetin, luteolin, quercetin 3-O-glycoside, apigenin, floridzin, and quercetin, which combined in an ethanolic extract, have been used by our research team, to demonstrate antifungal activity against *Fusarium* spp. [20]. Our research team also demonstrated that during murine acute toxicity measurements, even when administering *L. graveolens* Kunth stem extract concentrations as high as 2000 mg/Kg, the extract was classified in category 5 as nontoxic according to the guidelines set by the OECD-423 [21]. Furthermore, its potential use as a therapeutic agent against lung cancer, without affecting the viability of normal cells, was demonstrated using the same stem extract [22]. According to the latter and considering the cytotoxic issues that essential oils from leaves can present, the study of the effect of extracts from *L. graveolens* Kunth stem, and the resistant mechanism induced by its compounds aimed at combating fungal infections, becomes a relevant study.

In this study, we evaluated the effect of an ethanolic *L. graveolens* Kunth stem extract on both planktonic and biofilm-forming *C. albicans* cells. Furthermore, we assessed the modification in the cell wall (CW) proteome, focusing on the adherent subpopulation that persisted after treatment. This perspective adds originality to the study by examining the molecular features that support biofilm persistence and identify new therapeutic targets for developing antifungal strategies based on natural products in the face of increasing resistance and toxic effects associated with current antifungals.

## 2. Materials and Methods

### 2.1. Ethical Aspects

We used clinical *C. albicans* isolates (Table S1) provided by Hospital General 450 de Durango, Mexico. In all cases, the clinical isolates were obtained as part of routine diagnostic workup and used solely for research purposes without compromising the health or well-being of the donors. Since the samples were obtained as part of routine diagnostic procedures and did not contain any personal identifiable information, informed consent was not considered necessary by current regulations on microbiological research [23]. The Ethics Committee of Hospital General 450 de Durango reviewed and approved the protocol folio No. 34.

### 2.2. Collection of Plant Material

The plant material used in this study was the same as previously described [20]. Briefly, stems of *Lippia graveolens* Kunth were collected from the Oro Verde del Semidesierto Cooperative, Cuencamé, Durango. The specimen was recorded in the herbarium of the Interdisciplinary Research Center for Regional Development (CIIDIR-IPN), Durango, Mexico as *Lippia graveolens* Kunth (Vouchers 35542). Stems were ground to a particle size of 30 mesh with 8.5% moisture. The material was stored in sealed plastic bags and kept refrigerated at 4 °C until use.

### 2.3. Extract Preparation

The extracts used in this study were prepared according to the previously reported method [20]. Briefly, the stems of *Lippia graveolens* Kunth were macerated at room temperature (25 °C) with aqueous ethanol at concentrations of 80% and mass:solvent ratios of 1:30. The extraction was carried out for 24 h with stirring, followed by filtration, a second extraction with fresh solvent under the same conditions, and concentration using a rotary evaporator at 45 °C. The chemical composition of this extract has been previously characterized [20], revealing a profile enriched in naringenin (27%), taxofolin (21.1%), Caffeic acid (10.6%), eriodityol (18.1%), acacetin (0.7%), luteolin (8.6%), Quercetin 3-O-Glucoside (2.1%), Coumaric acid (3.8%), 2-hydroxybenzoic acid (1.7%), apigenin (1.5%), phrolidin (0.8%), quercetin (0.7%), Protocatechuic acid (0.9%), neohesperidin (0.7%), rutosidum (0.2%), quinic acid (0.3%), and 4-hydroxybenzoix acid (0.5%). The resulting aqueous extracts were lyophilized for subsequent use.

### 2.4. Strains and Culture Conditions

*C. albicans* isolates (Table S1) were routinely grown at 37 °C in a broth containing 1% yeast extract, 2% peptone, and 2% glucose (YPG). Eighteen strains of *C. albicans* from nosocomial candidemias used in this study correspond to clinical isolates kindly donated by the culture collection of Hospital General 450, Durango, Mexico. Species identification was confirmed by PCR amplification and sequencing of the internal transcribed spacer (ITS) region of the rDNA, according to the method described by White et al. [24]. Following molecular identification, strains were cryopreserved at −70 °C until use. The *C. albicans* ATCC 10231 strain was used as the reference strain.

### 2.5. Biofilm Formation Capacity of Clinical Isolates of C. albicans

The 18 *C. albicans* clinical isolates and the strain ATCC 10231 were grown in YPG medium at 37 °C and 120 rpm for 18 h. After this time, suspensions (10^6^ cells/mL) were prepared in RPMI 1640 medium and incubated in 96-well plates at 37 °C for 48 h. Subsequently, the wells were washed with phosphate buffer (0.1 M Na_2_HPO_3_•7H_2_O and NaH_2_PO_3_ at pH 7.2), stained with 0.4% crystal violet for 45 min, and washed with sterile distilled water. Subsequently, we added 95% ethanol to dry, and 100 μL of each sample was transferred to new plates to measure the optical density at 595 nm [25]. Biofilm formation was classified into four categories according to the optical density cutoff (ODc), calculated as the mean of the negative controls containing only RPMI 1640 medium (ODnc) plus three standard deviations (3 × SDnc). The categories were: negative biofilm (OD ≤ ODc), slightly positive (ODc < OD ≤ 2 × ODc), moderately positive (2 × ODc < OD ≤ 4 × ODc), and intensely positive (OD > 4 × ODc) [26]. Based on the above, *C. albicans* isolates were classified as high, moderate, low, or non-biofilm formers.

### 2.6. Evaluation of the Effect of L. graveolens Kunth Stem Extract on the Planktonic Growth of C. albicans

The *C. albicans* isolates were cultured independently in YPG medium at 37 °C, 120 rpm for 18 h. We then prepared suspensions of the *C. albicans* isolates using RPMI 1640 medium at a concentration of 10^3^ cells/mL. One hundred μL of each suspension was added to microtiter plate wells containing RPMI medium and extract, reaching a final volume of 200 μL in each well at *L. graveolens* Kunth stem extract concentrations of 2, 4, 8, 16, 32, and 64 mg/mL. Negative controls (0 mg/mL) were prepared using the same procedure, replacing *L. graveolens* Kunth stem extract with distilled water. Plates inoculated with the *C. albicans* isolates containing the different concentrations of the extract were incubated at 37 °C for 24 h. Due to the intense intrinsic coloration of the extract at higher concentrations, growth inhibition was subsequently evaluated visually. Fluconazole was used as a positive control according to CLSI guidelines [26].

### 2.7. Evaluation of the Effect of L. graveolens Kunth Stem Extract on Biofilm Formation in C. albicans Isolates

Biofilm-forming *C. albicans* isolate 2400H and strain ATCC 10231 were grown in YPG medium at 37 °C and 120 rpm for 18 h. Subsequently, we prepared suspensions in RPMI 1640 medium at a concentration of 10^6^ cells/mL. We added 100 μL of the suspensions to wells containing RPMI 1640 medium and *L. graveolens* Kunth stem extract, kindly provided by Cabral-Miramontes et al. [20], to a total volume of 200 μL and final extract concentrations of 0.16 μg/μL, 1.6 μg/ μL, and 16 μg/μL. After 24 h of incubation at 37 °C, non-adherent cells were removed by washing with phosphate buffer. Adherent cells were then mechanically scraped from each well and resuspended in phosphate buffer, and the optical density was measured at 600 nm. Negative controls were prepared using the same procedure, replacing *L. graveolens* Kunth stem extract with distilled water. The biofilm inhibition percentage was calculated according to the formula:

$$Biofilm\;inhibition\;percentage\;\left(\%\right)=\left(\frac{Optical\;density\;of\;controls-Optical\;density\;with\;extract}{Optical\;density\;of\;controls}\right)\times 100$$

### 2.8. Evaluation of the Effect of L. graveolens Kunth Stem Extract on Cell Wall Proteins of C. albicans 2400H Isolate

#### 2.8.1. Cell Lysis and Protein Extraction

Cell lysis of the highly biofilm-forming *C. albicans* 2400H isolate, in interaction with and without the extract, was carried out according to the method reported by [27]. After 24 h, the adherent cells were washed three times with PBS, scraped, transferred to an Eppendorf tube, and centrifuged at 3500 rpm for 5 min. The pellet cells were lysed using a homogenizer containing wash buffer (50 mM Tris-HCl, pH 7.5, supplemented with 1.0 mM phenylmethylsulfonyl fluoride (PMFS)) with SDS 0.5% and glass beads, performing five cycles of 1 min alternating with ice. The homogenate was centrifuged at 3500 rpm for 10 min at 4 °C, and the pellet with the CWPs was washed five times with wash buffer. Subsequently, an extraction buffer containing β-mercaptoethanol and 2% SDS was added according to the method reported by [28], and kept boiling for 10 min. After that, the homogenate was centrifuged at 3500 rpm for 15 min at 4 °C. The proteins in the supernatant were precipitated using 70% ethanol (1:3 *v*/*v*) and frozen at −20 °C for two hours. Finally, the proteins were centrifuged at 10,000 rpm for 15 min at 4 °C and resuspended in 100 μL of distilled water. We used the Bradford method to determine the concentration of the proteins extracted from *C. albicans* [29].

#### 2.8.2. Sample Preparation and Liquid Chromatography Coupled to Mass Spectrometry (LC-MS) Analysis

CWP’s extracted from biofilms of the clinical isolate 2400 H grown in the presence or absence of the extract were digested with 50 µg of total protein by Filter-Aided Sample Preparation (FASP) [30] with UA solution (8 M urea in 0.1 M Tris/HCl, pH 8) on passivated VivaSpin^®^ 500 filters (Cytiva, Marlborough, MA, USA) [31]. BSA was added to the concentrated eluates (Waters Corporation, Milford, MA, USA) as an internal standard, achieving 25 fmol/µL in 40 µL. We used mass spectrometry, applying the LC-MS analytical method described by [32] with some modifications. Chromatographic conditions included the use of an ACQUITY M-Class UPLC system, with mobile phase A (0.1% formic acid in water) and mobile phase B (0.1% formic acid in acetonitrile) under the following gradient: 7% B (0 min), 40% B (121.49 min), 85% B (123.15–126.46 min) and 7% B (129–130 min), at a constant flow rate of 400 L/min and a temperature of 45 °C. Spectral data were collected using a Synapt G2-Si mass spectrometer (Waters, Milford, MA, USA), using nanoelectrospray ionization (nanoESI) and ion mobility separation (IM-MS). Analysis was performed with a data-independent acquisition (DIA) approach through high-definition multiplexed MS/MS (HDMS^E^) mode. The ionization source parameters included 2.75 kV at the sampling capillary, 30 V at the cone and source offset, 70 °C at the source, 0.5 bar of nanoflow gas, and 150 L/h of purge gas. Chromatograms were recorded in positive mode within the *m*/*z* range 50–2000 with a scan time of 500 ms. No collision energy was applied for low-energy chromatograms, while high-energy chromatograms used a collision energy ramp of 19–55 eV to fragment precursor ions in the transfer cell. 

#### 2.8.3. Protein Database Search and Protein Quantification

The generated MS and MS/MS spectra contained in the raw files were processed, compared, and quantified using Progenesis QI for Proteomics v4.2 software (Nonlinear Dynamics, Newcastle, UK) [33], employing an inverted *Candida albicans* proteome (downloaded from UniProt, UP000005640) and the bovine serum albumin (BSA) fasta database (accession P02769). Parameters included trypsin as the cleavage enzyme with one missed cleavage, carbamidomethyl (C) as a fixed modification, and amidation (N-terminal), deamidation (N, Q), oxidation (M), and phosphorylation (S, T, Y) as variable modifications. The quantification was set by automatic tolerances for peptides and fragments, with the following minimum values: 2 fragments per peptide, five fragments per protein, one peptide per protein, and a false discovery rate (FDR) ≤ 4%. Absolute quantification was conducted using the three most reliable peptides per protein (Top 3), following the method of Silva et al. [34]. All identifications reached a confidence percentage ≥ 95% (Protein AutoCurate green). Calibration of the Synapt G2-S*i* spectrometer was carried out with [Glu1]-Fibrinopeptide ([M + 2H]^2+^ = 785.84261) with an accuracy ≤ 1 ppm.

Absolute protein quantification was performed according to the Top3 method reported by Arnaud-Franco et al. [35] following the next equation:

$$Top3=1/3\;\sum_{i=1}^{3}I_{i}$$

where the Top3 value is the mean of the MS signal response of the three most intense tryptic peptides. Using the Top3 value of the internal standard as well as of interest proteins, it is possible to determine the amount of protein in each sample using the following equation [33,34]:

$${fmol}_{A}=\frac{Top3_{A}}{Top3_{B}}({fmol}_{B})$$

where A is the protein of interest, B is the internal standard (BSA), and “fmol_B_” is the amount injected of internal standard.

The ratio was calculated by dividing the fmol value of each characterized protein in cells treated with the extract (treatment) by the corresponding value in untreated cells (control). All proteins considered differentially expressed had at least a ratio of ±0.585 (expressed as a base 2 logarithm) and a *p*-value ≤ 0.05.

For processing, we used the *Candida albicans* *.fasta database (from UniProt, UP000005640, with 79,052 protein sequences) supplemented with BSA (downloaded from UniProt, accession number: P02769). Bioinformatics analysis of total proteins and DEPs was performed using STRING-DB v12.0 (https://string-db.org/; accessed on 3 August, 2023). The resulting functional enrichments are biological process (gene ontology), cellular component (gene ontology), KEGG pathways, and subcellular localization (compartments).

### 2.9. Evaluation of the Effect of the Stem Extract on the Expression Profile of the FOB64_005423, ALS3, TSA1, and RBT1 Genes

Total RNA was extracted using the Trizol method from biofilms of *C. albicans* 2400H, with and without exposure to *L. graveolens* Kunth stem extract, after 24 h at 37 °C in liquid nitrogen. RNA was then treated with RNase-free DNase I (Thermo Fisher Scientific, Carlsbad, CA, USA), and ImProm-II^TM^ was used for cDNA synthesis following the manufacturer’s instructions. RT-qPCR reactions were developed with the next gene-specific primers FOB64_005423: Fwd 5′-TGCTGGTGGTGTTAATGGTG-3′ and Rev 5′-AGCTAAAGCCAAGACTGAGG-3′, *RBT1*: Fwd 5′-CAAAAACTAGTGCTCTCGTCT-3′ and Rev 5′-AGACCAATAATAGCAGCACCA-3′, *TSA1*: Fwd 5′-CTTGAGATTGTTGGAGGCTTT-3′ and Rev 5′-AGTATTCCTTGGATGCTTCTG-3′, *ALS3*: Fwd 5′-CAACCAATCTCAATCGCAATC-3′ and Rev 5’-ATCAAACCACATAACCAAGTAG-3′, using 100 ng of cDNA as template and the GoTaq^®^ qPCR master mix kit (Thermofisher). The RDN5.8 gene, a ribosomal protein [36], was used as a housekeeping gene. We used the 2^−ΔΔCt^ method to analyze the data using StepOne software v2.3 (Thermo Fisher Scientific, Waltham, MA. USA) to determine the relative expression of the selected genes.

### 2.10. Statistical Analysis

A completely randomized design was used and analyzed using a one-way analysis of variance (ANOVA) with a post hoc Bonferroni-Holm test. Graphs were created using OriginLab 2024^®^ software. Significance levels were defined as ns (*p* > 0.05), * (*p* ≤ 0.01), ** (*p* ≤ 0.001), and *** (*p* ≤ 0.0001) compared to the control groups. All experiments were performed in triplicate using two independent biological replicates, and the data were presented as mean ± standard deviation.

## 3. Results

### 3.1. Biofilm Formation Capacity in C. albicans Isolates

**Figure 1:**
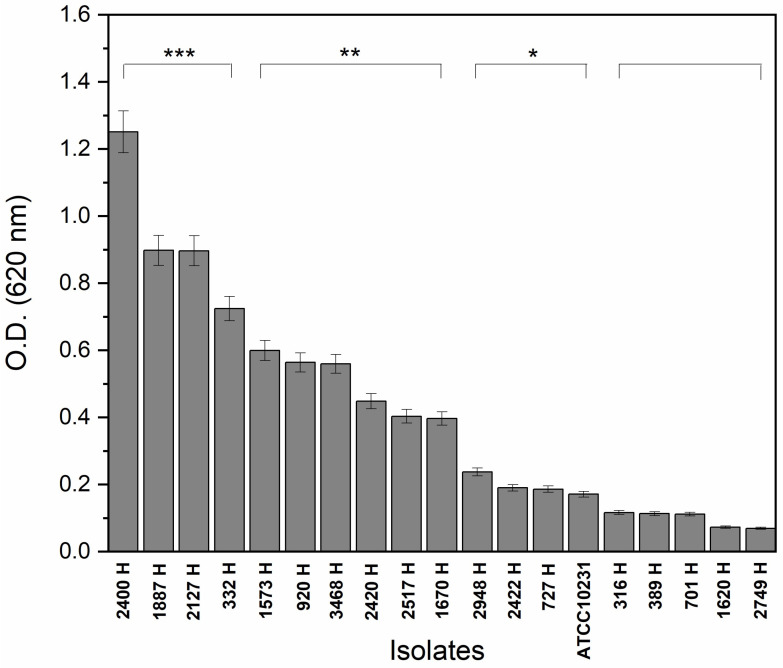
Biofilm formation capacity of *C. albicans* isolates. 18 clinical isolates and the ATCC 10231 strain were analyzed using the crystal violet method. The 2400H and 1887H isolates are the highest biofilm producers. Slightly positive * (ODc < OD ≤ 2 × ODc), moderately positive ** (2 × ODc < OD ≤ 4 × ODc), and intensely positive *** (OD > 4 × ODc).

To determine the biofilm formation capacity of the 18 *C. albicans* isolates, we used a spectrophotometric method (crystal violet). The optical density (OD) results are shown in Figure 1, wherein isolates 2400H and 2749H presented the highest and lowest biofilm formation capacity, respectively.

The *C. albicans* 2400H, 1887H, 2127H, 332H, 1573H, 920H, 3468H, 2420H, 2517H, and 1670H isolates presented significant differences compared to the reference strain ATCC 10231 used as the control. The isolates 2400H, 1887H, 2127H, and 332H showed more than 4-fold the OD than the control, the1573H, 920H, 3468H, 2420H, 2517H, and 1670H isolates showed an OD between 2 and 4-fold the OD than the control, while isolates 2948H, 2422H, 727H and the reference strain ATCC 10231 showed an OD higher than the control but lower than 2-fold. The isolates 316H, 389H, 701H, 1620H, and 2749H showed an OD mean value lower than or equal to that of control strain ATCC 10231.

Based on the classification criteria, from the 18 evaluated isolates of *C. albicans* clinical samples, 22.2% were high biofilm-forming (2400H, 1887H, 2127H, and 332H), 33.3% were moderate biofilm-forming (1573H, 920H, 3468H, 2420H, 2517H and 1670H), 16.6% low biofilm-forming (2948H, 2422H and 727H) and 27.9% non-biofilm-forming (316H, 389H, 701H, 1620H and 2749H) (Table 1). The reference strain ATCC 10231 was classified as a low biofilm-forming strain. Further experiments considered only high and moderate biofilm-forming *C. albicans* isolates from clinical origin and the reference strain ATCC 10231.

### 3.2. Effect of L. graveolens Kunth Stem Extract on the Planktonic Growth of Isolates of C. albicans

The biofilm formation capacity was evaluated in *C. albicans* isolates, and the *L. graveolens* Kunth 90% minimal inhibitory concentration (MIC_90_) was determined only in high (2400H, 1887H, 2127H, and 332H) and moderate (1573H, 920H, 3468H, 2420H, 2517H, and 1670H) biofilm-forming *C. albicans* isolates and the reference strain ATCC 10231. Table 2 shows that all the evaluated isolates exhibited an *L. graveolens* Kunth extract MIC_90_ of 16 mg/mL when incubated in RPMI media during planktonic growth. As a control, fluconazole was tested, and all isolates were susceptible.

### 3.3. Effect of L. graveolens Kunth Stem Extract on Biofilm Formation in C. albicans 2400H Isolate

**Figure 2:**
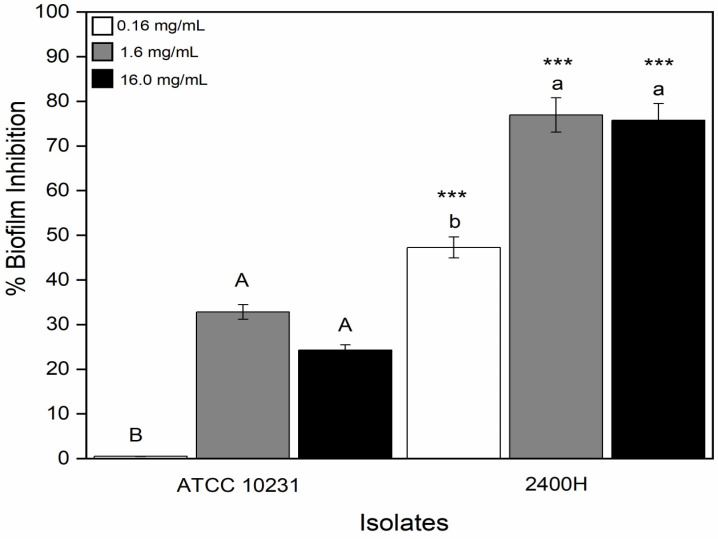
Biofilm formation inhibition in *C. albicans* with *L. graveolens* Kunth stem extract. The high biofilm-forming 2400H isolate displays a high inhibition of biofilm when exposed to 0.16 mg/mL, 1.6 mg/mL, and 16 mg/mL of stem extract. *** (*p* < 0.0001) compared to the reference strain ATCC 10231. Uppercase letters indicate statistical comparisons within ATCC 10231, and lowercase letters indicate statistical comparisons within 2400H. Identical letters denote no significant difference, while different letters indicate significant differences (*p* < 0.001).

To evaluate the effect of *L. graveolens* Kunth extract on biofilm formation, 2400H isolate and ATCC10231 strain were challenged with *L. graveolens* Kunth extract concentrations of 0.16 mg/mL, 1.6 mg/mL, and 16 mg/mL. The biofilm inhibition percentages are shown in Figure 2. At extract concentrations of 0.16 mg/mL, 1.6 mg/mL, and 16 mg/mL, 2400H isolate showed inhibition percentages of 47%, 77%, and 76%, respectively. ATCC10231 strain showed inhibition percentages of 0%, 33%, and 25% using the same concentrations. There were significant statistical differences in biofilm inhibition percentages between 2400H isolate and ATCC 10231 strain in all the tested concentrations. However, there were no statistical differences in biofilm inhibition percentages when comparing 1.6 mg/mL and 16 mg/mL concentrations in both strains, but there were when comparing 0.16 mg/mL concentration with 1.6 mg/mL and 16 mg/mL. The effect of the *L. graveolens* Kunth extract at 16 mg/mL was also evaluated in other biofilm-forming isolates. The results showed biofilm inhibition ranging from 18% to 77% (Figure S1).

### 3.4. Effect of L. graveolens Kunth Stem Extract in Cell Wall Proteins of C. albicans 2400H Isolate

**Figure 3:**
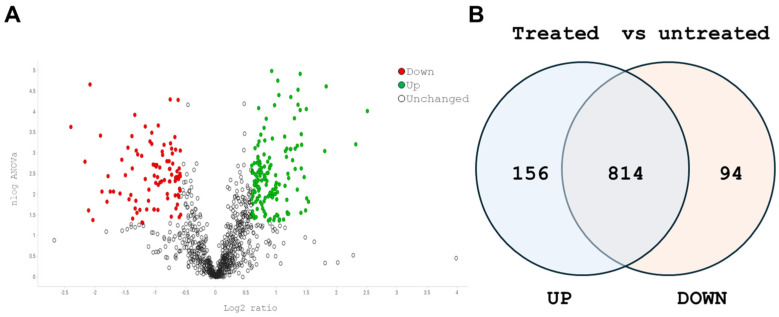
Differentially expressed CWPs in the 2400H isolate exposed to *L. graveolens* Kunth stem extract (1.6 mg/mL). (**A**) Volcano Plot showing the distribution of proteins according to changes in expression (log_2_ fold change) and statistical significance (−log_10_ *p*-value), with significantly regulated proteins highlighted in color. Red dots represent downregulated proteins, green dots represent upregulated proteins, and empty dots represent proteins that did not show significant changes relative to the control condition. (**B**) Venn diagram summarizes the total of proteins identified, indicating those upregulated, downregulated, and unchanged in treated cells compared to the untreated control.

To identify CWPs differentially produced in the cells that remained adherent after extract exposure, we analyzed the protein profile of the high biofilm-forming 2400H strain by comparing untreated and treated cells. The LC-MS analysis showed 1064 total proteins (Table S1) in 2400H isolate. Based on statistical significance (*p* < 0.05) and 0.5-fold higher or lower expression compared to control criteria, 156 and 94 proteins were up- and downregulated, respectively. As shown in Figure 3, the Volcano plot highlights proteins that were significantly up- or downregulated (Figure 3A), while the Venn diagram summarizes the total number of proteins identified, categorizing them as upregulated, downregulated, or unchanged compared to the untreated control (Figure 3B). Diverse subcellular localizations were identified in differentially expressed proteins, mainly CW-associated proteins (Figure S2). These results provide an overview of the proteomic response of the CW to the extract.

**Figure 4:**
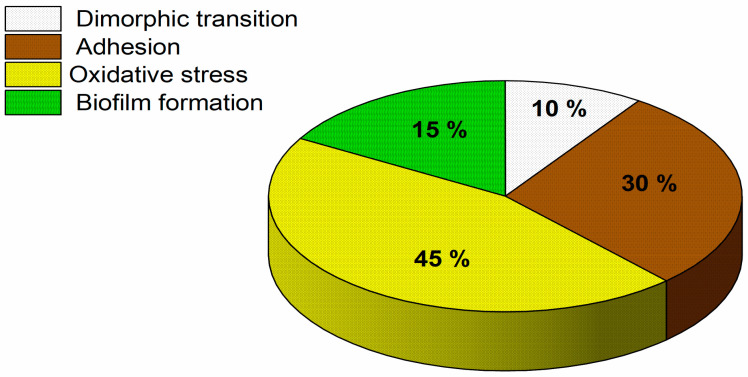
Classification of differentially expressed CWPs of *C. albicans* 2400H isolate exposed to *L. graveolens* Kunth stem extract.

A total of 40 CWPs differentially expressed were identified; the proteins were classified into dimorphic transition, adhesion, oxidative stress, and biofilm formation functions (Figure 4). The highest percentage was represented by oxidative stress proteins (45%), while dimorphic transition proteins represented the lowest (10%).

From the 40 CWPs identified, 25 proteins were up-regulated and 15 were down-regulated (Table 3). The GPI-anchored cell wall protein RBT1, the agglutinin-like protein 3 (ALS3), the Flocculin type 3 repeat family protein (FOB64_005423), and the thioredoxin peroxidase (TSA1) were relevant up-regulated proteins implicated in the different processes.

The up-regulated CWP protein RBT1, agglutinin-like protein 3 (Als3), flocculin type 3 repeat family protein (FOB64_005423), and thioredoxin peroxidase (Tsa1) have already been reported in previous research works as relevant proteins associated with dimorphic transition, adhesion, oxidative stress, and biofilm formation functions (Table 4).

### 3.5. Expression Levels of FOB64_005423, RBT1, ALS3, and TSA1 Genes of C. albicans 2400H Isolate Exposed to L. graveolens Kunth Stem Extract

**Figure 5:**
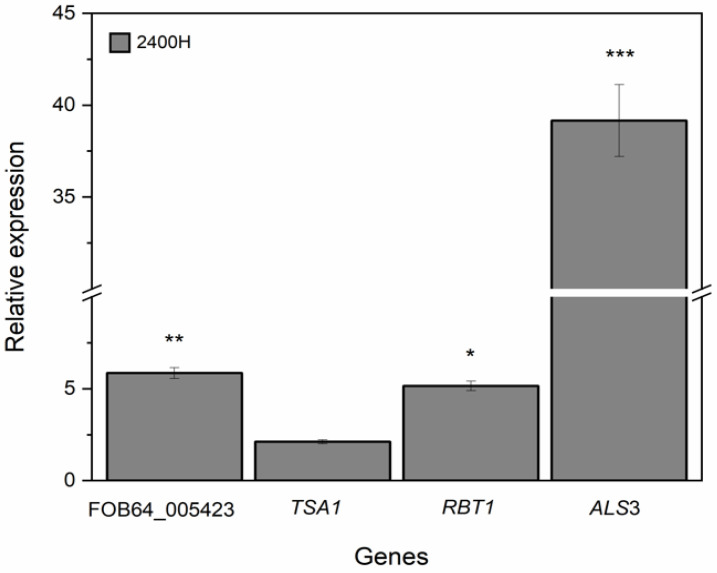
Relative expression profile of F0B64_005423, *TSA1*, *RBT1*, and *ALS3* genes of *C. albicans* 2400H isolate exposed to *L. graveolens* Kunth stem extract (1.6 mg/mL). The RDN5.8 gene was used as a housekeeping gene to normalize the data. Statistical analysis was performed with respect to cells without exposure to stem extract using one way-Anova with a post hoc Bonferroni-Holm test (Significance: * *p* < 0.01, ** *p* < 0.001, *** *p* < 0.0001).

To validate the obtained proteomic data of the 2400H isolate, we evaluated the expression levels using RT-qPCR on FOB64_005423, *RBT1*, *ALS3*, and *TSA1* genes coding for proteins reported in Table 4. Using the RDN5.8 as a housekeeping gene and the untreated cells as a control, the results showed that, *L. graveolens* Kunth stem extract positively regulated FOB64_005423, *RBT1*, *ALS3*, and *TSA1* genes in *C. albicans* 2400H isolate (Figure 5). The *RBT1* gene showed a relative expression of 6-fold higher than the control, while *TSA1* showed a relative expression of 2.5-fold higher than the control. Notably, the gene *ALS3* showed a relative expression 40-fold higher than the control.

## 4. Discussion

The results obtained in this research demonstrated that biofilm formation capacity among *C. albicans* clinical isolates differs from that of the reference strain ATCC 10231. The assay showed that after 24 h, ten *C. albicans* clinical isolates (2400H, 1887H, 2127H, 332H, 1573H, 920H, 3468H, 2420H, 2517H, and 1670H) exhibited higher biofilm production, and five (316H, 389H, 701H, 1620H, and 2749H) produced lower amounts of biofilm than the reference strain ATCC 10231. The latter results are consistent with studies where *C. albicans* isolates from candidiasis patients displayed a higher biofilm formation capacity compared to the reference strain ATCC 10231 [42]. A possible explanation for such results is that clinical experience has demonstrated that clinical isolates from fungal infections are more virulent and resistant to harsh conditions than collection strains. Previous works have found that *C. albicans* isolated from clinical mastitis exhibited a higher MIC_80_ than the reference strain ATCC 10231 when treated with *Floribundum mundy* [47].

The characterization of *L. graveolens* Kunth extracts has been reported in previous works, finding flavonoids such as naringenin, eriodyctiol, taxifolin, luteolin, and quercetin, and phenolic compounds such as hydroxyquinones [17,20,21]. Other research studies also reported the presence of different compounds, such as saponins and tannins [48]. The present work evaluated the effect of *L. graveolens* Kunth in fungal planktonic growth and biofilm formation, showing that all evaluated *C. albicans* isolates exhibited a MIC_90_ of 16 mg/mL. Cordoba et al. [9] determined the effect of an ethanolic extract of *L. junelliana* against strains of *Candida krusei*, *C. albicans*, *Candida glabrata*, and *Candida parapsilosis*, finding MIC’S_90_ of 0.00312 mg/mL, 0.40 mg/mL, 0.80 mg/mL, and 0.00312 mg/mL, respectively. Ruiz-Durán et al. [10] evaluated the antifungal activity of *L. alba* and *L. origanoides* extracts against *C. albicans*, *C. parapsilosis* and *C. auris*, reporting how *L. origanoides* extracts showed off MIC’S_50_ of 0.188 mg/mL, 0.141 mg/mL and 0.141 mg/mL for *C. albicans*, *C. parapsilosis*, and *C. auris,* respectively. They also reported a minimal inhibitory biofilm concentration (MIBC) of 0.188 mg/mL for *C. albicans* and *C. parapsilosis*, and 0.141 mg/mL for *C. auris*. More research works with different *Lippia* genus species as *L. citriodora*, *L. salviaefolia*, *L. velutina*, *L. balansae*, *L. lasiocalycina* and *L. lupulina*, have demonstrated antifungal effect against *C. parapsilosis*, *C. albicans*, *C. krusei*, and *Cryptococcus neoformans,* reporting MIC’S among 0.156 mg/mL and 0.250 mg/mL [11,13].

In our study, despite concentrations of *L. graveolens* Kunth extract being higher than MICs_90_ reported in extracts from other *Lippia* species, it must be considered that the extract used in this work was obtained from the plant stem, a discarded by-product of the essential oil industry, with a lower content of bioactive compounds. Furthermore, in order to increase the antifungal effect of the *L. graveolens* Kunth stem extract, future research related to bioformulations through emulsions or nanoemulsions could be considered, since it has been demonstrated that these formulations could increase the bioactivity of *Lippia* genus species extracts [12,49,50].

Some of the works mentioned above have reported the effect of natural extracts on germinal tube formation and yeast-to-hyphae transition. After adhesion, the next steps in biofilm production are germinal tube formation and hyphal elongation. In our work, compared to controls, the *L. graveolens* Kunth stem extract concentrations reported in this study reduced biofilm formation between 47% and 77% in the *C. albicans* isolate 2400H. Notably, a similar inhibitory effect was observed even at 1.6 mg/mL, a concentration tenfold lower than 16 mg/mL, which may be considered physiologically and therapeutically manageable [21,22]. At the MIC_90_, the extract completely inhibited planktonic cells but only reduced biofilms by 77%, reflecting the higher tolerance of biofilm-associated cells due to protective factors such as the extracellular matrix and reduced compound penetration [51].

A recent work [40], using 28 oleic acid-like saponins extracted from *Pulsatilla chinensis*, reported that *C. albicans* diminished its adhesion capacity and biofilm formation in the presence of concentrations between 6.25 µg/mL and 100 µg/mL of the saponin pulchinenoside B3, also reducing the expression of genes associated with yeast-to-hyphae transition. The diminishment of biofilm formation and preformed biofilm degradation has also been reported using extracts from *Mentha* species, showing a negative regulation in genes associated with adhesion [42]. Kim et al. [39] reported a decay in the expression levels of genes associated with adhesion and biofilm formation in *C. albicans* using hydroxyquinones, compounds contained in extracts of plants such as *L. graveolens* Kunth. A *L. origanoides* leaf extract has also demonstrated its effectiveness not only in *C. albicans* biofilm formation diminishment but also in the reduction in biofilm formation of different microorganisms such as *Lactobacillus rhamnosus* and *Streptococcus mutans*, which are associated with caries formation. However, despite the effectiveness of inhibiting biofilm formation, the extract obtained from the leaves of this plant showed cytotoxic levels similar to the compound chlorhexidine, a compound that causes undesirable effects during its prolonged use [19].

To better understand the effect induced by *L. graveolens* Kunth stem extract over *C. albicans* and the possible response mechanism of the fungus to this challenge, a proteomic LC-MS analysis was carried out in an enriched fraction from the CW obtained from the treatment-tolerant adherent cells. Through this analysis, a total of 250 CWPs were identified, but only 40 were differentially expressed. The differentially expressed proteins were classified into four functions: dimorphic transition, adhesion, oxidative stress, and biofilm formation. Different research works have evaluated through proteomic approaches the effect of plant extracts such as *Cytrus hystrix* and *Murraya koenigii* over *C. albicans*, finding a differential production of proteins associated with diverse functions such as mitochondrial matrix, cellular and metabolic processes, transition, antioxidant activities, adhesion, filamentous growth, etc. [38,52]. In the same sense, carrying out a proteomic study through LC-MS/MS to evaluate the effect in a whole population of *C. albicans* (adherent and non-adherent cells) exposed to β-citronellol, a monoterpene naturally found in the plant *Citrus hystrix*, Buakaew et al. [38] found that from the main 126 proteins identified, altered in response to β-citronellol, the CWPs Als2p, Rbt1p, and Pga4 and the oxidative stress response proteins Sod1p, Gst2p, and Ddr48p, contrary to our work, were downregulated. Muthamil et al. [52], working with loosely adherent planktonic cells, reported a possible mechanism of oleic acid obtained from the plant *Murraya koenigii* against *C. albicans*. Using a MALDI-TOF/TOF proteomic analysis using differentially expressed 2D SDS-PAGE spots, they established that oleic acid inhibited proteins associated with yeast-to-hyphae transition, biofilm formation, and fungal adhesion capacity, and modified the amount of ergosterol in the inner cell membrane. Later results were complemented through RT-qPCR, confirming that the genes that code for these proteins were negatively regulated. The results in the present work demonstrated a higher production of cell surface proteins associated with adhesion, oxidative stress, dimorphic transition, and biofilm formation coded by FOB64_005423, *TSA1*, *RBT1*, and *ALS3* genes. The same results were further obtained by RT-qPCR, confirming opposite results to previous works [38,39,48,52]. The differences in our results compared to previous works were expected, since our proteomic analysis specifically targeted *C. albicans* cells that remained adherent after 24 h of exposure to the *L. graveolens* Kunth extract, allowing the identification of wall-associated proteins potentially involved in biofilm persistence, discarding non-adherent cells or susceptible subpopulations. However, analysis of the fraction that remained adherent allows the identification of determinants associated with tolerance and biofilm persistence. The overexpression of FOB64_005423, *TSA1*, *RBT1*, and *ALS3* genes could be a compensatory stress response mechanism in the *C. albicans* 2400H isolate, induced by the extract, since dimorphic fungi have shown adaptation to changes in growth conditions and external factors to remodel or repair their surface, in response to environmental stress using different stress response pathways [53,54]. However, this possible mechanism needs to be confirmed with further experiments. Future studies should consider separate or comparative proteomic analyses of both adherent and non-adherent *C. albicans* cells after treatment, to capture the full spectrum of cellular responses to the extract. Moreover, the present findings may inform subsequent efforts aimed at identifying specific inhibitors or therapeutic combinations that more effectively target biofilm persistence.

While the present study provides molecular and proteomic evidence supporting the antibiofilm activity of the *L. graveolens* stem extract and its impact on CW–associated proteins, future studies could further expand these findings by incorporating complementary approaches. In particular, metabolic activity assays and advanced microscopic analyses (e.g., confocal or electron microscopy) would be valuable to correlate the observed proteomic changes with biofilm viability, architecture, and structural organization. The integration of these methodologies may offer additional insights into the phenotypic consequences of extract exposure and contribute to a more comprehensive understanding of its effects on *C. albicans* biofilms.

## 5. Conclusions

The results demonstrate that the ethanolic *L. graveolens* Kunth stem extract exhibits significant antifungal activity against *C. albicans* in both its planktonic and biofilm forms. Furthermore, these findings emphasize the potential of *L. graveolens* Kunth stem as an alternative source of bioactive compounds with antifungal activity, supporting their relevance in strategies aimed at controlling biofilm formation. The CW proteome revealed proteins associated with fungal dimorphic transition, adhesion, oxidative stress, and biofilm formation. These findings shed light on key tolerance mechanisms in adherent biofilm-forming cells.

## Figures and Tables

**Table 1:** Classification of *C. albicans* isolates from clinical origin based on biofilm formation capacity.

Biofilm Forming	Isolate	Number of Isolates	Percentage (%)
High	22400H, 1887H, 2127H, and 332H	4	22.2
Moderate	1573H, 920H, 3468H, 2420H, 2517H, and 1670H	6	33.3
Low	ATCC 10231, 2948H, 2422H, and 727H	3	16.6
Non-forming	316H, 389H, 701H, 1620H, and 2749H	5	27.9

**Table 2:** Sensitivity to *L. graveolens* Kunth stem extract in biofilm-forming isolates of *C. albicans* from clinical origin.

Biofilm Forming	Isolates	Extract-MIC_90_ (mg/mL)	Fluconazole-MIC_90_ (µg/mL)
High	2400H	16	<0.125
High	1887H	16	0.125
High	2127H	16	<0.125
High	332H	16	0.125
Moderate	1573H	16	0.25
Moderate	920H	16	0.25
Moderate	3468H	16	<0.125
Moderate	2420H	16	0.125
Moderate	2517H	16	<0.125
Moderate	1670H	16	0.5
Low	ATCC 10231	16	0.25

**Table 3:** Differentially expressed CWPs of *C. albicans* 2400H isolate exposed to *L. graveolens* Kunth stem extract.

Uniprot No. Access	ENA Gene ID	Protein Putative Function	Log_2_ Fold Change	*p*
A0A8H6F377	FOB64_004736	Lysophospholipase	2.50	0.0000
A0A8H6F4R1	*RBT1*	Cell wall protein RBT1	2.32	0.0006
A0A8H6BX16	*CHT3*	Chitinase 3	1.41	0.0003
A0A8H6BUX2	*GAM1*	Glucoamylase 1	1.40	0.0000
A0A8H6BYS4	*ALS3*	Agglutinin-like protein 3	1.39	0.0000
A0A8H6BUN5	*NCP1*	NADPH-cytochrome P450 reductase	1.17	0.0007
A0A8H6BT95	FOB64_005423	Flocculin type 3 repeat family protein	1.14	0.0062
A0A8H6BQY9	FOB64_006659	Thioredoxin reductase	1.02	0.0000
A0A8H6BYJ9	FOB64_004041	Ribosomal protein S10	0.97	0.0073
A0A8H6BXS5	FOB64_002993	Nascent polypeptide-associated complex subunit beta	0.93	0.0004
A0A8H6BQW6	*CHT2*	Chitinase 2	0.902	0.0399
A0A8H6F6X4	FOB64_000863	Translationally controlled tumor protein homolog	0.867	0.0030
A0A8H6F124	*MP65*	Cell surface mannoprotein MP65 domain protein	0.83	0.0057
A0A8H6F3U2	FOB64_002340	Arginase	0.82	0.0057
A0A8H6BWF6	FOB64_004094	Phosphotransferase	0.81	0.0301
A0A8H6F501	*ADO1*	Adenosine kinase	0.80	0.0004
A0A8H6F4L7	*TSA1*	Peroxiredoxin TSA1-A	0.77	0.0220
A0A8H6F3P9	*VPS21*	Vacuolar protein sorting-associated protein 21	0.75	0.0029
A0A8H6BXS5	FOB64_002993	Nascent polypeptide-associated complex subunit alpha	0.72	0.0073
A0A8H6BZX4	FOB64_001976	Glycerol-3-phosphate dehydrogenase [NAD(+)]	0.72	0.0108
A0A8H6F2S3	*HSP78*	Heat shock protein 78_mitocondrial	0.70	0.0074
A0A8H6BU09	FOB64_004742	Adenine phosphoribosyltransferase	0.68	0.0064
A0A8H6BQY7	swoH	Nucleoside diphosphate kinase	0.60	0.0257
A0A8H6C397	FOB64_002507	14-3-3 domain-containing protein	0.60	0.0029
A0A8H6C3Z6	*RPL19B*	Ribosomal protein L19	−0.59	0.0098
A0A8H6F362	FOB64_003210	6-phosphogluconate dehydrogenase	−0.59	0.0082
A0A8H6BUX0	*IPP1*	Inorganic diphosphatase	−0.64	0.0073
A0A8H6F4H4	*RPL4B*	60S ribosomal protein L4-B	−0.66	0.0027
A0A8H6BZ37	*RPS6A*	40S ribosomal protein S6	−0.68	0.0008
A0A8H6BYQ8	*PGI1*	Glucose-6-phosphate isomerase	−0.75	0.0004
A0A8H6C486	*MET6*	5-methyltetrahydropteroyltriglutamate--homocysteine S-methyltransferase	−0.77	0.0000
A0A8H6F4K9	FOB64_002438	Ribosomal protein L10	−0.97	0.0015
A0A8H6BRV2	FOB64_006313	Phosphoglycerate mutase	−1.05	0.0019
A0A8H6BU78	FOB64_006449	Cytochrome P450	−1.11	0.0003
A0A8H6F012	FOB64_005613	Methionine adenosyltransferase	−1.16	0.0141
A0A8H6F3P4	FOB64_004035	Aconitate hydratase_mitocondrial	−1.31	0.0042
A0A8H6BVM4	FOB64_006072	Catalase family protein	−1.34	0.0001
A0A8H6BWI4	FOB64_005268	Flavodoxin-like domain-containing protein	−1.69	0.0085
A0A8H6C4N6	*FDH1*	Formate dehydrogenase	−1.91	0.0003
A0A8H6BZT5	*SOD5*	Cell surface Cu-only superoxide dismutase 5	−2.40	0.0002

ENA: European Nucleotide Archive.

**Table 4:** Relevant CWPs differentially expressed in *C. albicans* 2400H exposed to *L. graveolens* Kunth stem extract.

Name	Function	Log_2_ Fold Change	References
Cell wall protein, Rbt1	GPI-anchored cell wall protein required for virulence, mating efficiency, biofilm formation, and dimorphism.	2.32	[37,38,39]
Agglutinin-like protein 3, Als3	Crucial role in the adhesion stage, biofilm formation, and dimorphism.	1.39	[37,40,41,42,43,44]
Flocculin type 3 repeat family protein, FOB64_005423	Adhesion of the pathogen to host tissue cells or abiotic surfaces such as catheters and implants.	1.14	[43,44]
Peroxiredoxin Tsa1	Ability to protect biomolecules from oxidative damage.	0.77	[45,46]

## Data Availability

The original contributions presented in this study are included in the article and Supplementary Material. Further inquiries can be directed to the corresponding author.

## Supplementary Materials

The following supporting information can be downloaded at: https://www.mdpi.com/article/10.3390/pathogens15020216/s1, Figure S1: Biofilm formation inhibition in *C. albicans* isolates with *L. graveolens* Kunth stem extract; Figure S2: Subcellular localization of differentially expressed proteins; Table S1: Clinical and reference *C. albicans* isolates used in this study; Table S2: Total CWPs quantified.

## References

[1] Pu S., Niu S., Zhang C., Xu X., Qin M., Huang S., Zhang L. (2017). Epidemiology, antifungal susceptibilities, and risk factors for invasive candidiasis from 2011 to 2013 in a teaching hospital in southwest China. J. Microbiol. Immunol. Infect..

[2] Cavalheiro M., Teixeira M.C. (2018). *Candida* biofilms: Threats, challenges, and promising strategies. Front. Med..

[3] Finkel J.S., Mitchell A.P. (2011). Genetic control of *Candida albicans* biofilm development. Nat. Rev. Microbiol..

[4] Pereira R., dos Santos-Fontenelle R.O., de Brito E.H.S., de Morais S.M. (2021). Biofilm of *Candida albicans*: Formation, regulation and resistance. J. Appl. Microbiol..

[5] Nobile C.J., Johnson A.D. (2015). *Candida albicans* biofilms and human disease. Annu. Rev. Microbiol..

[6] Ji H.F., Li X.J., Zhang H.Y. (2009). Natural products and drug discovery. EMBO Rep..

[7] Abdul-Wahab S.M., Jantan I., Haque M.A., Arshad L. (2018). Exploring the leaves of *Annona muricata* L. as a source of potential anti-inflammatory and anticancer agents. Front. Pharmacol..

[8] Hadisaputri Y.E., Habibah U., Abdullah F.F., Halimah E., Mutakin M., Megantara S., Diantini A. (2021). Antiproliferation activity and apoptotic mechanism of soursop (*Annona muricata* L.) leaves extract and fractions on MCF7 breast cancer cells. BCTT.

[9] Cordoba S., Vivot W., Szusz W., Albo G. (2019). Antifungal activity of essential oils against *Candida* species isolated from clinical samples. Mycopathologia.

[10] Ruiz-Duran J., Torres R., Stashenko E.E., Ortiz C. (2023). Antifungal and antibiofilm activity of Colombian essential oils against different *Candida* strains. Antibiotics.

[11] Funari C.S., Gullo F.P., Napolitano A., Carneiro R.L., Mendes-Giannini M.J.S., Fusco-Almeida A.M., Piacente S., Pizza C., Silva D.H.S. (2012). Chemical and antifungal investigations of six *lippia* species (Verbenaceae) from Brazil. Food Chem..

[12] Prado J.C.S., de Aguiar F.L.L., Prado G.M., do Nascimento J.F., de Souza N.V., Barbosa F.C.B., Lima D.M., Rodrigues T.H.S., Bessa N.U.C., Abreu F.O.M.S. (2024). Development and characterization of nanoemulsions containing *Lippia origanoides* Kunth essential oil and their antifungal potential against *Candida albicans*. J. Appl. Microbiol..

[13] Ghasempour M., Omran S.M., Moghadamnia A.A., Shafiee F. (2008). Effect of aqueous and ethanolic extracts of *Lippia citriodora* on *Candida albicans*. Electron. Physician.

[14] Pozatti P., Scheid L.A., Spader T.B., Atayde M.L., Santurio J.M., Alves S.H. (2008). In vitro activity of essential oils extracted from plants used as spices against fluconazole-resistant and fluconazole-susceptible *Candida* spp.. Can. J. Microbiol..

[15] Freire I.A., Bueno-Silva B., Galvao L.C.D.C., Duarte M.C.T., Sartoratto A., Figueira G.M., de Alencar S.M., Rosalen P.L. (2015). The effect of essential oils and bioactive fractions on *Streptococcus mutans* and *Candida albicans* biofilms; a confocal analysis. Evid. Based. Complement. Alternat. Med..

[16] Gutiérrez-Grijalva E.P., Antunes-Ricardo M., Acosta-Estrada B.A., Gutiérrez-Uribe J.A., Heredia J.B. (2019). Cellular antioxidant activity and in vitro inhibition of α-glucosidase, α-amylase and pancreatic lipase of oregano polyphenols under simulated gastrointestinal digestion. Food Res. Int..

[17] Frías-Zepeda M.E., Rosales-Castro M. (2021). Effect of extraction conditions on the concentration of phenolic compounds in Mexican oregano (*Lippia graveolens* Kunth) residues. Rev. Chapingo Ser. Cienc. For. Ambiente.

[18] Morshedloo M.R., Alireza S.S., Nazeri V., Maggi F., Craker L. (2018). Essential oil profile of oregano (*Origanum vulgare* L.) populations grown under similar soil and climate conditions. Ind. Crop. Prod..

[19] Loaiza-Oliva M., Morales-Uchima S.M., Puerta-Suárez J., Mesa-Arango A.C., Martínez Pabón M.C. (2023). *Lippia origanoides* derivatives in vitro evaluation on polymicrobial biofilms: *Streptococcus mutans*, *Lactobacillus rhamnosus* and *Candida albicans*. Arch. Oral Biol..

[20] Cabral-Miramontes J.P., Martínez-Rocha A.L., Rosales-Castro M., López-Rodríguez A., Meneses-Morales I., Del Campo-Quinteros E., Herrera-Ocelotl K.K., Gándara-Moreno G., Velázquez-Huizar S.J., Ibarra-Sánchez L. (2024). Antifungal Activity of Mexican Oregano (*Lippia graveolens* Kunth) Extracts from Industrial Waste Residues on *Fusarium* spp. in Bean Seeds (*Phaseolus vulgaris* L.). Agriculture.

[21] Frías-Zepeda M.E., Rosales-Castro M., Escalona-Cardoso G.N., Paniagua-Castro N. (2022). Ethanolic extract of *Lippia graveolens* stem reduce biochemical markers in a murine model with metabolic syndrome. Saudi J. Biol. Sci..

[22] Frías-Zepeda M.E., Ibarra-Berumen J., Ordaz-Pichardo C., Rosales-Castro M. (2022). Cytotoxic activity of ethanolic extracts of *Lippia graveolens* HBK leaves and stem against lung cancer cell line SK-LU-1. Blacpma.

[23] Secretaría de Salud Reglamento de la ley General de Salud en Materia de Investigación Para la Salud. Diario Oficial de la Federación, México 2014. https://www.dof.gob.mx/.

[24] White T., Bruns T., Lee S., Taylor J. (1990). Amplification and direct sequencing of fungal ribosomal RNA genes for phylogenetics. Acad. Pre. Inc..

[25] Nikawa H., Jin C., Makihira S., Egusa H., Hamada T., Kumagai H. (2003). Biofilm formation of *Candida albicans* on the surfaces of deteriorated soft denture lining materials caused by denture cleansers in vitro. J. Oral Rehabil..

[26] Shrief R., Zaki M.E.S., El-Sehsah E.M., Ghaleb S., Mofreh M. (2019). Study of antifungal susceptibility, virulence genes and biofilm formation in *Candida albicans*. Open Microbiol. J..

[27] Ruiz-Baca E., Leyva-Sánchez H., Calderón-Barraza B., Esquivel-Naranjo U., López-Romero E., López-Rodríguez A., Cuéllar-Cruz M. (2019). Identification of proteins in *Sporothrix schenckii sensu stricto* in response to oxidative stress induced by hydrogen peroxide. Rev. Iberoam. Micol..

[28] Félix-Contreras C., Alba-Fierro C.A., Ríos-Castro E., Luna-Martínez F., Cuéllar-Cruz M., Ruiz-Baca E. (2020). Proteomic analysis of *Sporothrix schenckii* cell wall reveals proteins involved in oxidative stress response induced by menadione. Microb. Pathog..

[29] Bradford M.M. (1976). A rapid and sensitive method for the quantitation of microgram quantities of protein utilizing the principle of protein-dye binding. Anal. Biochem..

[30] Wisniewski J.R., Zougman A., Nagaraj N., Mann M. (2009). Universal sample preparation method for proteome analysis. Nat. Methods.

[31] Erde J., Loo R.R.O., Loo J.A. (2014). Enhanced FASP (eFASP) to increase proteome coverage and sample recovery for quantitative proteomic experiments. J. Proteome Res..

[32] Ríos-Castro E., Souza G.H.M.F., Delgadillo-Álvarez D.M., Ramírez-Reyes L., Torres-Huerta A.L., Velasco-Suárez A., Tapia-Ramírez J. (2020). Quantitative proteomic analysis of MARC-145 cells infected with a Mexican porcine reproductive and respiratory syndrome virus strain using a label-free based DIA approach. J. Am. Soc. Mass Spectrom..

[33] Li H., Rose M.J., Tran L., Zhang J., Miranda L.P., James C.A., Sasu B.J. (2009). Development of a method for the sensitive and quantitative determination of hepcidin in human serum using LC-MS/MS. J. Pharmacol. Toxicol. Methods.

[34] Silva J.C., Gorenstein M.V., Li G.Z., Vissers J.P., Geromanos S.J. (2006). Absolute quantification of proteins by LCMSE: A virtue of parallel MS acquisition* S. Mol. Cell. Proteom..

[35] Arnaud-Franco G., Ríos-Castro E., Velasco-Suárez A., García-de León F.J., Beltrán L.F., Carbajal-Saucedo A. (2023). Venom comparisons of endemic and micro-endemic speckled rattlesnakes *Crotalus mitchellii*, *C. polisi* and *C. thalassoporus* from Baja California Peninsula. Toxicon.

[36] Li Q.Q., Skinner J., Bennett J.E. (2012). Evaluation of reference genes for real-time quantitative PCR studies in *Candida glabrata* following azole treatment. BMC Mol. Biol..

[37] Liu H. (2001). Transcriptional control of dimorphism in *Candida albicans*. Curr. Opin. Microbiol..

[38] Buakaew W. (2022). Proteomic analysis reveals proteins involved in the mode of action of β-citronellol identified from *citrus hystrix* DC. leaf against *Candida albicans*. Front. Microbiol..

[39] Kim Y.G., Lee J.H., Park S., Khadke S.K., Shim J.J., Lee J. (2022). Hydroquinones including tetrachlorohydroquinone inhibit *Candida albicans* biofilm formation by repressing hyphae-related genes. Microbiol. Spectr..

[40] Tan J., Zhang Z., Zheng D., Mu Y., Cao B., Yang J., Han L., Huang X. (2024). Structure-activity relationship and biofilm formation-related gene targets of oleanolic acid-type saponins from *Pulsatilla chinensis* against *Candida albicans*. Bioorg. Chem..

[41] Li L., Wei M.P., Yu H., Xie Y.F., Guo Y.H., Cheng Y.L. (2023). Antifungal activity of *Sapindus* saponins against *Candida albicans*: Interruption of biofilm formation. J. Herb. Med..

[42] Norouzi N., Alizadeh F., Khodavani A., Jahangiri M. (2021). Antifungal activity of menthol alone and in combination on growth inhibition and biofilm formation of *Candida albicans*. J. Herb. Med..

[43] Willaert R.G. (2018). Adhesins of yeasts: Protein structure and interactions. J. Fungi.

[44] Willaert R.G., Kayacan Y., Devreese B. (2021). The Flo adhesin family. Pathogens.

[45] Gutiérrez-Escobedo G., Hernández-Carreón O., Morales-Rojano B., Revuelta-Rodríguez B., Vázquez-Franco N., Castaño I., De Las Peñas A. (2020). *Candida glabrata* peroxiredoxins, Tsa1 and Tsa2, and sulfiredoxin, Srx1, protect against oxidative damage and are necessary for virulence. Fungal Genet. Biol..

[46] Ramírez-Quijas M.D., López-Romero E., Cuéllar-Cruz M. (2015). Proteomic analysis of cell wall in four pathogenic species of *Candida* exposed to oxidative stress. Microb. Pathog..

[47] Ksouri S., Djebir S., Bentorki A.A., Gouri A., Hadef Y., Benakhla A. (2017). Antifungal activity of essential oils extract from *Origanum floribundum* Munby, *Rosmarinus officinalis* L. and *Thymus ciliatus* Desf. against *Candida albicans* isolated from bovine clinical mastitis. J. Mycol. Med..

[48] Cortés-Chitala M.D.C., Flores-Martínez H., Orozco-Ávila I., León-Campos C., Suárez-Jacobo Á., Estarrón-Espinosa M., López-Muraira I. (2021). Identification and quantification of phenolic compounds from Mexican oregano (*Lippia graveolens* HBK) hydroethanolic extracts and evaluation of its antioxidant capacity. Molecules.

[49] Herrera-Rodríguez S.E., López-Rivera R.J., García-Márquez E., Estarrón-Espinoza M., Espinoza-Andrews H. (2018). Mexican Oregano (*Lippia graveolens*) essential oil-in-water emulsions: Impact of emulsifier type on the antifungal activity of *Candida albicans*. Food Sci. Biotechnol..

[50] Gil G.A., Kakuda L., Tonani L., von Zeska Kress M.R., Oliveira W.P. (2025). Surfactant-driven effects on the antifungal activity of *Lippia origanoides* Kunth essential oil encapsulated in lipid-based nanosystems. ACS Omega.

[51] Singh R., Kumari A., Kaur K., Kaur R. (2018). Relevance of antifungal penetration in biofilm-associated resistance of *Candida albicans* and non-*albicans Candida* species. J. Med. Microbiol..

[52] Muthamil S., Prasath K.G., Priya A., Precille P., Pandian S.K. (2022). Global proteomic analysis deciphers the mechanism of action of plant derived oleic acid against *Candida albicans* virulence and biofilm formation. Sci. Rep..

[53] Ortiz-Ramírez J.A., Cuéllar-Cruz M., López-Romero E. (2022). Cell compensatory responses of fungi to damage of the cell wall induced by calcofluor white and congo red with emphasis on *Sporothrix schenckii* and *Sporothrix globosa*. A review. Front. Cell. Infect. Microbiol..

[54] Iyer K.R., Robbins N., Cowen L.E. (2022). The role of *Candida albicans* stress response pathways in antifungal tolerance and resistance. iScience.

