# Supplementary material for: Cell Wall Proteome of Candida albicans Reveals Proteins Associated with Tolerance to Antibiofilm Activity of a Lippia graveolens Kunth Stem Extract
Source: Pathogens. 2026 Feb 14;15(2):216. doi: 10.3390/pathogens15020216 (PMC12942757; doi:10.3390/pathogens15020216)
Supplement: Supplementary file 1 [file pathogens-15-00216-s001.zip › Table S1.pdf]

Table S1. Clinical and reference *C. albicans* isolates used in this study.

**Isolate code**	**Species**	**Origin**	**Clinical source**	**Identification method**
1670-H	*C. albicans*	Clinical	Blood culture	ITS sequencing
2422-H	*C. albicans*	Clinical	Blood culture	ITS sequencing
2517-H	*C. albicans*	Clinical	Blood culture	ITS sequencing
1620-H	*C. albicans*	Clinical	Blood culture	ITS sequencing
2127-H	*C. albicans*	Clinical	Blood culture	ITS sequencing
920-H	*C. albicans*	Clinical	Blood culture	ITS sequencing
332-H	*C. albicans*	Clinical	Blood culture	ITS sequencing
2420-H	*C. albicans*	Clinical	Blood culture	ITS sequencing
316-H	*C. albicans*	Clinical	Blood culture	ITS sequencing
389-H	*C. albicans*	Clinical	Blood culture	ITS sequencing
701-H	*C. albicans*	Clinical	Blood culture	ITS sequencing
727-H	*C. albicans*	Clinical	Blood culture	ITS sequencing
1573-H	*C. albicans*	Clinical	Blood culture	ITS sequencing
1887-H	*C. albicans*	Clinical	Blood culture	ITS sequencing
2749-H	*C. albicans*	Clinical	Blood culture	ITS sequencing
2400-H	*C. albicans*	Clinical	Blood culture	ITS sequencing
2948-H	*C. albicans*	Clinical	Blood culture	ITS sequencing
3468-H	*C. albicans*	Clinical	Blood culture	ITS sequencing
ATCC 10231	*C. albicans*	Reference strain	-	ATCC certified strain
