# Supplementary material for: Cell Wall Proteome of Candida albicans Reveals Proteins Associated with Tolerance to Antibiofilm Activity of a Lippia graveolens Kunth Stem Extract
Source: Pathogens. 2026 Feb 14;15(2):216. doi: 10.3390/pathogens15020216 (PMC12942757; doi:10.3390/pathogens15020216)
Supplement: Supplementary file 1 [file pathogens-15-00216-s001.zip › Table S2.pdf]

Table S2. Total CWPs quantified.

No.	Accesion	Anova (p)	-Log (FDR)
1	A0A8H6C1A2	0.74481059	0.05211432
2	A0A8H6BT95	0.00621946	1.14673323
3	A0A8H6C1T2	0.69528328	0.04881808
4	A0A8H6C300	0.3250273	0.12227019
5	Q3MPX7;A0A8H6F4S5	0.347285	0.27282335
6	A0A8H6BT91	0.48547626	0.0717692
7	A0A8H6BU87;A0A8H6BWS8;A0A8H6BV34	0.82140019	0.03164136
8	A0A8H6C061	0.00370899	0.68113553
9	A0A8H6F5V1	0.14851703	-0.19438375
10	A0A8H6BU46	0.1537537	0.15212776
11	A0A8H6BSH4	0.63560914	0.08110336
12	A0A8H6F1X2	0.63061215	-0.16091158
13	A0A8H6F4U9	0.712254	0.10335143
14	REVERSE1900	0.74793249	-0.13383161
15	A0A8H6BX35	0.7884726	0.03661589
16	A0A8H6BV36	0.00768001	0.57867562
17	A0A8H6F4L7	0.02202889	0.77340315
18	A0A8H6F165	0.61780414	-0.06190789
19	A0A8H6C633	0.00660127	0.70556095
20	A0A8H6F504	0.34942879	0.16795644
21	A0A8H6EZS6	0.05356156	0.40940245
22	A0A8H6C3A1	0.00066102	1.34897996
23	A0A8H6BZ79	0.001229	0.69497448
24	A0A8H6BW79	0.01266789	0.59834448
25	A0A8H6BS49	0.69041329	-0.15606888
26	A0A8H6F0E5	0.72989734	0.06620806
27	A0A8H6C395	0.03042543	0.44462655
28	A0A8H6BW35	0.55240378	0.05370087
29	A0A8H6F0N4	0.03433012	0.55933162
30	A0A8H6C5X8	0.0554444	0.24893627
31	A0A8H6BWK4	0.00090269	1.8040694
32	A0A8H6C372	0.05542939	0.36320371
33	A0A8H6BWH1	0.01347226	0.54984948
34	A0A8H6C2F2	0.08245846	0.41198163
35	A0A8H6BVT8	0.00243224	1.31583036
36	G1UA71	0.29245441	0.15908682
37	A0A8H6C444	0.01411169	0.47673347
38	A0A8H6BXS5	0.00044115	0.93635958
39	A0A8H6BWW5	0.98047112	0.00135748
40	A0A8H6C5N4	0.80615087	-0.08928594
41	A0A8H6BZV0	0.00182118	-0.3218749
42	A0A8H6F2S3;A0A8H6F3E1	0.00740309	0.70942306

43	A0A8H6F4L5	0.08883493	0.25891607
44	A0A8H6BTT2	0.79518647	-0.09493309
45	A0A8H6F0G0	0.50247163	0.19127275
46	A0A8H6BU53	0.90363807	-0.06856893
47	A0A8H6BS29	0.41589094	0.07221752
48	G1UAY2	0.00163463	0.72907876
49	A0A8H6BX63	0.01508933	0.29624493
50	A0A8H6C655	0.79647025	-0.03332792
51	A0A8H6C3N3	0.33580712	-0.11433831
52	A0A8H6BS39	0.895847	0.22769656
53	A0A8H6BUW9;A0A8H6BY59	0.75173381	0.03537461
54	A0A8H6F347;A0A8H6F530	0.00057643	-0.75072756
55	A0A8H6BU35	0.03505514	-0.25446147
56	A0A8H6F4G0	0.91496495	-0.01733558
57	A0A8H6F3Z7	0.35080511	0.15154868
58	A0A8H6F3Y8	0.16064823	0.21925025
59	A0A8H6BWC3	0.00546483	0.66609493
60	A0A8H6BYS1	0.96614614	-0.00738221
61	A0A8H6BWF6	0.01215647	0.81668001
62	A0A8H6C3C1	0.0073656	0.72417879
63	A0A8H6C3K1	0.98969783	-0.00419202
64	A0A8H6C5X6	0.06434472	0.3805862
65	A0A8H6C139	0.34305116	0.27053266
66	A0A8H6F2U2	0.8664929	0.00607696
67	A0A8H6BX86	0.54793052	-0.08906517
68	A0A8H6C3E7	0.79128231	0.03232362
69	A0A8H6C5L4	0.00453197	0.70134821
70	P02769	0.79716198	0.15286326
71	A0A8H6F0V4;A0A8H6F0F7	0.41925058	-0.13103345
72	A0A8H6BVU5;A0A8H6BZR7	0.00077143	1.31253767
73	A0A8H6F4H4	0.00082905	-0.66752944
74	A0A8H6BYW3	0.01984988	0.2037769
75	A0A8H6C0B0	0.0028867	0.72077141
76	A0A8H6BUP1	0.68623798	0.07197467
77	A0A8H6F2N4	0.89891739	-0.05326819
78	A0A8H6F3Z3	0.03357448	0.51995319
79	A0A8H6F4B7	0.00984339	0.54168777
80	A0A8H6F332	0.07561741	-0.32332593
81	A0A8H6BRV9;A0A8H6F6E0	4.39E-05	1.24419859
82	A0A8H6F6A1	0.1863062	-0.18974212
83	A0A8H6F4N1	0.56497746	0.06536865
84	A0A8H6BWZ3	0.78547606	-0.08350976
85	A0A8H6C387	0.00426286	-0.61920191
86	A0A8H6F3Y6	8.62E-05	1.50096751
87	A0A8H6C0P1	0.03005373	-0.35753959
88	A0A8H6F5J9	0.13772237	0.21590672
89	A0A8H6C4F3	0.10025045	0.3314993

90	A0A8H6BQY9	1.74E-05	1.02567942
91	A0A8H6BZE2;A0A8H6F152	0.00492097	-0.89956005
92	A0A8H6BYA0	0.55620054	-0.2017881
93	A0A8H6F0T0	0.33476965	-0.31145676
94	A0A8H6BR90	0.71630725	0.05744899
95	A0A8H6BXX0	0.00197217	0.46703526
96	A0A8H6F5S8	0.00404046	1.20801156
97	A0A8H6C0C8	0.00107813	0.80178642
98	A0A8H6BXT1	0.00177173	0.72111266
99	A0A8H6C431	0.88403659	0.0201948
100	A0A8H6F6C8	0.02612072	0.37148227
101	A0A8H6F0K9;A0A8H6F118	0.68126697	-0.19140339
102	A0A8H6C4Y1	0.80534248	-0.07303701
103	A0A8H6F1M1	0.14389276	0.5477875
104	A0A8H6F3U2	0.03013064	0.82551491
105	A0A8H6EZZ4;A0A8H6F5F8	0.43492387	0.14278162
106	A0A8H6F0N5	0.00487278	-0.76017954
107	A0A8H6F4N0	0.77061463	-0.03933468
108	A0A8H6C107	0.17798847	0.15469623
109	A0A8H6C3I0	0.48243338	0.09502158
110	A0A8H6C0F8	0.97630231	-0.01002022
111	A0A8H6BZ90	0.0037693	-0.65548069
112	A0A8H6F5Q4	0.00468133	-0.4163253
113	A0A8H6BWE6	0.01033376	0.90703403
114	A0A8H6C0E2	0.20010097	-0.30794209
115	A0A8H6F1R7	0.7678422	0.03687624
116	A0A8H6F501	0.00049469	0.8099273
117	G1UA43	0.66729833	0.18045429
118	A0A8H6BVK4	0.00793021	-0.54488019
119	A0A8H6BSZ8	0.00182783	-0.53170282
120	A0A8H6BZU1	0.9188471	-0.01218701
121	A0A8H6BU22	0.965161	-0.00021859
122	A0A8H6F4Z7	0.12057616	-0.15800688
123	G1UAI4	0.07401348	0.4515964
124	A0A8H6BZK2	0.56469205	-0.11768495
125	A0A8H6BZD4	0.94967425	0.00284329
126	A0A8H6F0S0	0.0311458	-0.58101272
127	A0A8H6BVN2	0.0031999	-0.59261084
128	A0A8H6BYJ9	0.0073763	0.97776538
129	A0A8H6BYQ8	5.01E-05	-0.75837127
130	A0A8H6F353	0.00758533	0.44131873
131	G1UAZ6	0.17495137	0.05424767
132	A0A8H6BV80;A0A8H6BZC3	0.18426767	-0.19899602
133	G1UAP3;A0A8H6C033;A0A8H6F622	0.6537095	0.07694922
134	A0A8H6F3T7	0.91353767	0.01037434
135	A0A8H6F4S4	0.86581552	0.0013358
136	A0A8H6C4E2;A0A8H6F591	0.03047816	0.35601298

137	A0A8H6F443	0.66410164	-0.19109746
138	A0A8H6BVC3	0.00178511	0.73131436
139	A0A8H6F5T7	0.26914641	-0.42108065
140	A0A8H6EZT7	0.00036271	0.74719945
141	A0A8H6BUX0	0.00272764	-0.64207736
142	A0A8H6C2N6	0.05500027	0.39246724
143	A0A8H6C5D5	0.4001006	-0.10217916
144	A0A8H6BV76	0.45427594	0.41677238
145	A0A8H6BU68;G1UAV9	0.00173307	0.6403215
146	A0A8H6F1X4	0.0047742	0.65350066
147	A0A8H6BZJ3	0.41067281	-0.16040913
148	A0A8H6BRY3	0.92606451	-0.07283591
149	A0A8H6F6B7	0.63802111	-0.08984255
150	A0A8H6F3D2	0.81794154	0.04307458
151	A0A8H6BV49	0.63678065	0.1029962
152	A0A8H6F4E7	0.04066995	-0.35297569
153	A0A8H6C0S0	0.01189679	0.45516157
154	A0A8H6BUP3	0.25970245	0.14976612
155	REVERSE2441	0.29497261	-0.28752694
156	A0A8H6F3P2	0.02789185	0.31906438
157	A0A8H6F0L2	0.07252172	0.46187993
158	A0A8H6F7A7;A0A8H6F638;A0A8H6F6M3	0.65034692	0.11065527
159	A0A8H6BWR7	0.01143333	-0.47777878
160	A0A8H6C1I7	0.11088349	0.4734564
161	A0A8H6BXM8	0.18864381	-0.26418359
162	A0A8H6EZQ5	0.02338072	-0.3284535
163	A0A8H6F259	0.38562929	0.2318518
164	A0A8H6F1B1;A0A8H6F598	0.71541665	-0.19308525
165	A0A8H6F1R9	0.00040114	1.14258248
166	A0A8H6C449	0.00291109	0.69996571
167	A0A8H6C290	0.01556646	0.74081592
168	A0A8H6F2A4	0.00290674	0.61623184
169	A0A8H6F362	0.00734761	-0.59834288
170	A0A8H6F4A6;A0A8H6F4Y9	0.53833465	-0.20587161
171	A0A8H6BTM2	0.02096661	-0.47306406
172	A0A8H6BY98	0.96760204	0.05131826
173	A0A8H6BZ37	0.00040423	-0.68042449
174	A0A8H6F1S8	0.00083867	0.4655951
175	G1UA53	0.03497394	0.5982504
176	A0A8H6F1S5	0.7005359	-0.07878316
177	A0A8H6BUY5;A0A8H6BU36	0.40400013	0.17803225
178	A0A8H6F630	0.00940181	-0.52727597
179	A0A8H6F6L1	0.09444304	-0.30260822
180	A0A8H6C4V3	0.46286262	0.03450038
181	A0A8H6EZQ8	0.33026025	0.19827289
182	A0A8H6BVY8	0.00386912	-0.66336651

183	A0A8H6BTR7	0.50315001	0.39533927
184	A0A8H6BT25;A0A8H6BRD5	0.0202834	-0.4644285
185	A0A8H6F3G2	0.00014789	0.83423294
186	A0A8H6BRD7	0.98607668	-0.08119013
187	A0A8H6C357	0.89265103	0.0291254
188	A0A8H6F2S2	0.00033557	0.47782392
189	A0A8H6C196	0.23474292	0.53804231
190	A0A8H6F678	0.74888464	-0.1774347
191	A0A8H6BTX3	0.05291472	-0.4332611
192	A0A8H6F6D6	0.09242032	0.19606986
193	A0A8H6C2R2	0.14780639	-0.55277602
194	A0A8H6BWZ1	0.43672274	0.06815729
195	A0A8H6BUN5;A0A8H6F1I8	0.00078281	1.17723699
196	A0A8H6BTK0	0.02261113	-0.34521637
197	A0A8H6BST6	0.00445818	-0.85334922
198	A0A8H6BZ02	0.80820846	-0.089228
199	A0A8H6C2K3	3.40E-05	1.07303406
200	A0A8H6BUP8	0.00340281	0.98988368
201	A0A8H6BYL8	0.09812465	0.40573458
202	A0A8H6BV15	0.47016976	0.08729621
203	A0A8H6F1X9	0.0015903	-0.80723298
204	A0A8H6BUC8	0.00256549	0.72259947
205	A0A8H6BW57	0.05148202	-0.27437328
206	A0A8H6F462	0.00513971	-0.92397969
207	A0A8H6F6G2	0.21109477	-0.23098303
208	A0A8H6F4Z3	0.83083181	0.07297123
209	A0A8H6BT49	0.22567898	-0.14538171
210	A0A8H6C1H7	0.06352091	0.24497101
211	A0A8H6C3L2;A0A8H6F697	0.00211863	0.80570681
212	A0A8H6BVV9	0.01245654	0.48483955
213	A0A8H6BZA9;A0A8H6C0N5	0.26375304	-0.20116213
214	A0A8H6BWZ6	0.00075374	0.65175165
215	A0A8H6F5K4	0.06279964	-0.38709145
216	G1UAD0	0.02191189	0.24739565
217	A0A8H6F026	0.03563607	-0.60617912
218	A0A8H6F4K9	0.00193567	-0.97608865
219	A0A8H6F0Y7	0.42654735	0.1187043
220	A0A8H6BW47	0.00380754	0.41753607
221	A0A8H6F0G9	0.07771714	0.39930177
222	A0A8H6F5N7	0.19900776	0.1481332
223	G1UAM5;A0A8H6C194	0.77946229	0.04588347
224	A0A8H6F4C2	0.80242581	-0.07529374
225	G1UAZ3	0.010957	0.61605294
226	A0A8H6F0H8	0.44555036	-0.25558782
227	A0A8H6F5X0;O60036;G1UAC5	0.02579439	-0.39656238
228	A0A8H6BZ32	0.00197203	-1.00504901
229	A0A8H6BUE1	6.88E-05	0.9723747

230	A0A8H6C2R3	0.1819509	0.20602903
231	A0A8H6C1R9	0.10777284	0.27453145
232	A0A8H6C337	0.01130072	-0.57813992
233	A0A8H6BZX4	0.01082132	0.72412587
234	A0A8H6F4I1	0.00946591	0.92542843
235	A0A8H6BYF6	0.75925685	0.18510351
236	A0A8H6BTS1	0.01760125	-0.50436747
237	A0A8H6F1E3	0.00289898	0.71130825
238	A0A8H6BV10	0.18391604	0.30875196
239	A0A8H6BWP6	0.03464803	-0.73492534
240	A0A8H6F5C3	0.55278596	-0.09920181
241	A0A8H6F2P9	0.09723488	0.24738715
242	A0A8H6C4F1	0.55438799	-0.07282158
243	A0A8H6BW14	0.00496314	-0.495582
244	A0A8H6BRQ0	0.06238525	0.43436379
245	A0A8H6C0E5	0.87663735	0.03502009
246	A0A8H6BZ84	0.00811439	0.63469824
247	A0A8H6C1T3	0.3609883	-0.25089794
248	A0A8H6BY41	0.9560757	0.11110176
249	A0A8H6BVW0	0.00123598	1.17206525
250	A0A8H6BRP1	0.00260009	0.60156856
251	A0A8H6BRQ2	0.12109504	-0.52189422
252	A0A8H6F0V0	0.00117853	-1.22996162
253	A0A8H6F2G8	0.00079177	1.23532172
254	Q9P4W0	0.01521495	0.66118784
255	A0A8H6BU09	0.00649204	0.68339021
256	A0A8H6BW97;A0A8H6C696	0.12398616	0.20738964
257	A0A8H6BUD2	0.91287035	0.01059079
258	A0A8H6F4A7	0.85799306	-0.20409226
259	A0A8H6F4F2;A0A8H6F698	0.03370227	0.27928828
260	REVERSE3189	0.04482143	0.38461355
261	A0A8H6BY24	1.03E-05	0.92780537
262	A0A8H6C397	0.0029887	0.60431693
263	A0A8H6BUD9	0.83680357	0.01979135
264	A0A8H6F1C8	0.35253357	0.10598918
265	A0A8H6C4U1	0.00854177	-0.43768073
266	A0A8H6C1A5	0.18536611	-0.83577898
267	A0A8H6BZ44;A0A8H6F4L4	0.0006281	1.42103304
268	A0A8H6BU58	0.00236851	0.88772862
269	A0A8H6F650	0.57669267	-0.0524197
270	A0A8H6BZC0	0.09363969	-0.59423333
271	A0A8H6BQY7	0.02573625	0.60574842
272	A0A8H6BR52	0.00809424	0.70930151
273	A0A8H6C026	0.00077858	-0.73358178
274	A0A8H6F5K0	0.00237265	-0.60897404
275	A0A8H6F509;G1UA77;A0A8H6C0L5	0.29568636	2.27430399
276	A0A8H6BUW6	0.72028087	0.06985136

277	A0A8H6C486	0.00155171	-0.77079914
278	A0A8H6BU78	0.01419088	-1.11429009
279	A0A8H6C1I5	0.19711363	-0.30604497
280	A0A8H6F5V7	0.0693939	-0.40130145
281	A0A8H6F4X4	0.02228925	0.68877154
282	A0A8H6BZZ5	0.0069883	1.13539418
283	A0A8H6BUV6	0.30099455	-0.66157612
284	A0A8H6C3P5	0.53417019	0.21876752
285	A0A8H6C3P1;A0A8H6C5P7	0.00470238	0.84777404
286	A0A8H6BRT8	0.31972547	0.10703882
287	A0A8H6BW27	0.01253062	0.68641137
288	A0A8H6C0U1	0.00322986	-0.48289155
289	A0A8H6BX28	5.14E-05	-0.62223893
290	A0A8H6C271	0.0067601	-0.3999644
291	A0A8H6F3R6	0.02523756	0.28736522
292	A0A8H6F355	0.30315999	-0.31010957
293	A0A8H6BU39	0.0542819	0.69569636
294	A0A8H6BUX8;A0A8H6BX46	0.05117127	0.47099053
295	A0A8H6BX74	0.05904738	0.17143942
296	A0A8H6F4S3;A0A8H6C253	0.11382679	0.27261661
297	A0A8H6F5B2	0.00352336	0.73651706
298	A0A8H6C635	0.92886215	-0.06095974
299	A0A8H6BT56;A0A8H6C1Q7	0.006221	0.52172341
300	A0A8H6EZR9	0.14731744	0.23945988
301	A0A8H6C5F9	0.00199352	0.59719541
302	A0A8H6BUX5	0.58439573	0.18217879
303	A0A8H6BZS6	0.45876905	0.25688238
304	A0A8H6C2H5;A0A8H6C383	0.00488824	0.63917251
305	A0A8H6F2C1	0.00427215	0.8139662
306	Q3MPJ3	0.02697577	0.45610006
307	A0A8H6BSV3	0.2261707	-0.43676893
308	A0A8H6BVQ2	0.07225626	-0.37420241
309	A0A8H6F151	0.00110317	-0.87979011
310	A0A8H6BUV8;A0A8H6C5M8;A0A8H6F2S5	0.13951851	-0.20554328
311	A0A8H6F5D7	0.97015755	0.00022585
312	A0A8H6C259	0.0125213	0.37176712
313	Q9HF92;A0A8H6C2D4	0.32878156	0.12534637
314	A0A8H6BZ88	0.63911625	-0.08856057
315	A0A8H6BXG9	0.05823042	0.77562577
316	A0A8H6F2T6	0.94136687	0.00835058
317	A0A8H6C652	0.58390243	-0.07454531
318	G1UAM9	0.00343189	0.79653475
319	A0A8H6C479	0.00052331	0.71289247
320	A0A8H6BWT8	0.01020872	0.5570524
321	A0A8H6BT44	0.92331556	0.11619029
322	A0A8H6BX02	0.82337466	0.05998378

323	A0A8H6BUG0	0.08401456	-1.06050147
324	A0A8H6BZ56	0.01068837	-0.67243948
325	A0A8H6F5P2;A0A8H6F6F8	0.20710042	0.40896609
326	A0A8H6BVU6;A0A8H6F3A6	0.79664422	0.08649853
327	A0A8H6BXE3	0.03032187	0.59011717
328	A0A8H6F354	0.67656857	-0.19404434
329	A0A8H6F012	0.00428571	-1.16645077
330	A0A8H6C2P7	0.00608033	0.64346951
331	A0A8H6C4G7	0.03114993	0.48015896
332	A0A8H6BVF5	0.50773225	0.19665603
333	A0A8H6F6C0	0.41313492	-0.14798322
334	REVERSE2683	0.5015975	-0.24614178
335	A0A8H6C3T4;A0A8H6F737;A0A8H6F5I1;A0A8H6C3Y9	0.05940432	0.20260772
336	A0A8H6F1A1	0.00462517	0.75821213
337	A0A8H6C2U3	0.00317852	1.19640882
338	A0A8H6C2T3	0.01330264	0.69161946
339	A0A8H6BRX3	0.08614325	-0.5360631
340	A0A8H6F2L8	0.00351352	-1.02138973
341	A0A8H6BSG2	0.134327	-0.16945692
342	A0A8H6BWR2	0.37701568	0.12593789
343	A0A8H6BS67	0.00172366	0.68661228
344	A0A8H6BX13	0.10080514	-0.47303044
345	A0A8H6BR57	0.012517	-0.30088013
346	A0A8H6C044	0.01494685	0.48985096
347	A0A8H6F2Q7;G1UAH0	0.4109284	-0.22793256
348	A0A8H6BUB1	0.49617662	0.1239588
349	A0A8H6C4M6	0.00682455	0.99402579
350	A0A8H6F0Q9	0.03820649	-0.34403848
351	A0A8H6BVY7;A0A8H6BXB0	0.02662861	0.48774483
352	A0A8H6F2D4	0.51491293	0.16074251
353	G1UAF4	0.12363189	0.33106717
354	A0A8H6C4Q1;A0A8H6C3U5;A0A8H6C5Z4	0.06260927	0.76192981
355	A0A8H6C0Y7	0.57022508	0.22098237
356	A0A8H6BY74	0.99539197	0.44406669
357	A0A8H6C4S3	0.59805384	0.07508385
358	A0A8H6C2U8	0.01253877	1.50740656
359	A0A8H6BWI6	0.03712199	0.54094395
360	A0A8H6C1B9	0.0447047	0.42552751
361	A0A8H6C634	0.52822735	0.09969779
362	A0A8H6F311	0.19307095	-0.52382693
363	A0A8H6F5F6	0.00846883	0.56652514
364	A0A8H6C229;G1UAT0	0.19256068	-0.487551
365	A0A8H6BWL0;A0A8H6BVB7	0.74668044	-0.12209652
366	A0A8H6C367	0.29463371	0.1632199
367	A0A8H6C3Z6	0.00820565	-0.59453984

368	A0A8H6BS07	0.01563639	0.44816966
369	A0A8H6C016	0.01225258	0.37143406
370	A0A8H6C5K5	0.68821545	-0.04900919
371	A0A8H6BSW3	0.04393504	0.35608653
372	A0A8H6C0G4	0.06006107	0.93621064
373	A0A8H6BR15	0.00533623	1.00346506
374	A0A8H6F1T8	0.03248471	-0.41525485
375	A0A8H6C4M3	0.01502809	0.55931557
376	M1T132	0.0155975	0.54772685
377	A0A8H6BUR4	0.12148447	0.29315623
378	A0A8H6C349	0.04337824	0.54149603
379	A0A8H6F4H9	0.00672431	0.7095871
380	A0A8H6BZC1	0.10345842	0.68072278
381	A0A8H6BYU5	0.01805782	0.49480414
382	A0A8H6F743	0.20740875	0.37382301
383	A0A8H6F0M2	0.0373675	0.63736268
384	A0A8H6C509	0.03552046	-0.31454108
385	A0A8H6F2Y3	0.0728612	-0.3005319
386	A0A8H6C1K7	0.00585707	0.64051008
387	A0A8H6BRT6	0.00251845	-0.35791548
388	A0A8H6BY10	0.45481373	-0.08812357
389	A0A8H6C0G6	0.27736469	-0.39941529
390	A0A8H6C0M3;A0A8H6F4G7	0.01601718	0.5602918
391	A0A8H6F6X4	0.00303531	0.86777632
392	A0A8H6F0K3	0.00998667	0.60640379
393	G1UAL7	0.36712301	0.17790557
394	A0A8H6F5L1	0.06071706	0.53779938
395	A0A8H6F042	0.23060284	0.19560419
396	A0A8H6BWQ8	0.03374032	0.80867067
397	A0A8H6C4I9	6.45E-05	0.47344752
398	A0A8H6BRK4	0.93382735	0.02682313
399	A0A8H6BT40	0.23658184	-0.60691459
400	A0A8H6BT18	0.09658049	0.40232794
401	A0A8H6C3A3;A0A8H6BZC6	0.00195442	0.72235105
402	A0A8H6BW82	0.22708832	0.1093867
403	A0A8H6C544	0.00973327	0.98552574
404	A0A8H6F4E3	0.12868138	-0.45273069
405	A0A8H6C2C4;A0A8H6C4G3	0.44875643	0.15495479
406	A0A8H6F4L3	0.21326029	-0.21889503
407	A0A8H6BUY3	0.55266276	0.10088969
408	A0A8H6C014	0.33674223	0.30840714
409	G1UAR0	0.06452011	-0.54162562
410	A0A8H6C1X1	0.55170038	-0.14026669
411	A0A8H6F588;A0A8H6F6V7;A0A8H6F6M5	0.70507423	0.52940199
412	A0A8H6BTK6	0.00074272	-1.45251685
413	A0A8H6C533;A0A8H6C2P5	0.0033095	-0.73074612

414	A0A8H6BUN2;A0A8H6C3V7	0.00289117	0.74213031
415	A0A8H6C5R4	0.00203021	0.36656098
416	A0A8H6BRH8	0.69920214	0.06634515
417	A0A8H6BX57	0.81300535	0.1195456
418	A0A8H6F124	0.00578974	0.83449211
419	A0A8H6F1D8	0.31464063	0.18782242
420	G1UAL4	0.00089007	1.15510789
421	A0A8H6BZX6	0.1930231	0.15236203
422	A0A8H6F3L0	0.33821633	-0.26376109
423	A0A8H6C5A0	0.90841459	-0.01872677
424	A0A8H6C4X7;A0A8H6C3P7	0.0272963	-0.39052345
425	A0A8H6F3W9	0.00830815	0.34813243
426	Q9P8X1	0.39084474	-0.3804258
427	A0A8H6C350	0.23116774	-0.44823155
428	A0A8H6F6W2	0.00978935	-0.80420231
429	A0A8H6F017	0.01109348	0.31626951
430	A0A8H6F6F7	0.94727305	0.04285174
431	A0A8H6BXF0	0.00065259	0.69676847
432	A0A8H6BTW2	0.20981426	0.2767803
433	A0A8H6BY13	0.94040712	0.01046467
434	A0A8H6C446	0.62609517	0.17670149
435	A0A8H6BV23	0.58154412	0.23220827
436	A0A8H6C0Q2	0.76897672	-0.02548028
437	A0A8H6BWX5	0.38248723	0.26896286
438	A0A8H6BT81	0.16793326	0.14698537
439	A0A8H6C3M3	0.18657195	0.24583713
440	A0A8H6BZL1;A0A8H6C3I4	0.52486115	-0.19109111
441	A0A8H6C355	0.0262799	0.5674902
442	A0A8H6C220	0.01963532	0.29401648
443	A0A8H6BX70	0.56427973	0.07448543
444	G1UAW4;A0A8H6C0V0	0.07167316	0.35273326
445	A0A8H6F6Y6	0.02267394	0.51438072
446	A0A8H6BX85	0.04371133	0.52565549
447	A0A8H6F286	0.25625756	-0.16771694
448	A0A8H6C1A1;A0A8H6BWS5	0.06233715	0.25043336
449	A0A8H6BTY1	0.57443017	0.14330221
450	A0A8H6F1U2	3.96E-05	1.04659944
451	A0A8H6C129	0.08866382	0.39978284
452	A0A8H6C4K9	0.26713957	0.19486107
453	A0A8H6C5N2	0.31591777	0.99104033
454	A0A8H6BRN7;A0A8H6F0S7	0.05116011	0.51043029
455	A0A8H6BXF4	0.03436125	0.52422456
456	A0A8H6BVK0	0.00772223	1.35262766
457	A0A8H6F6Q0	0.49774875	0.18747898
458	A0A8H6BYR6	0.07063278	-0.24982416
459	A0A8H6F4C8	0.1605382	0.292108
460	A0A8H6C0T9;A0A8H6BYW0	0.04974866	0.36236567

461	A0A8H6C5S8	0.74875978	-0.12532917
462	A0A8H6F3X2	0.73407805	0.23119656
463	A0A8H6BT47	0.92989128	-0.01175014
464	A0A8H6C5Y9	0.30126541	-0.19711712
465	A0A8H6BV09	6.75E-05	1.36423731
466	A0A8H6C2W6	0.49174616	0.31123433
467	A0A8H6C3P6	0.40961241	0.16556577
468	A0A8H6F2W1	0.57581968	-0.07234366
469	A0A8H6C4C5	0.00308264	0.84544944
470	A0A8H6F632	0.17053727	0.35573943
471	A0A8H6C1D3	0.31100707	0.27340274
472	A0A8H6F692	0.5106427	0.04500471
473	A0A8H6BR88;A0A8H6BSU5	0.52178575	-0.11626023
474	A0A8H6BUD0	0.13925842	0.54440963
475	A0A8H6C5Z3	0.00174057	0.68984845
476	A0A8H6F0C3	0.00871798	0.65278303
477	A0A8H6BVK2	0.03144734	-0.2577179
478	A0A8H6BQW6	0.039906	0.90213329
479	A0A8H6C2M9	0.02583385	0.79797274
480	A0A8H6BZV1	0.31770925	0.29220034
481	A0A8H6BUP5;A0A8H6BYK4;A0A8H6BXU3	0.0827278	-0.75913448
482	A0A8H6EZV8	0.709643	0.04491449
483	REVERSE4448	0.76458988	0.16828387
484	G1U9Y3	0.01099817	0.90795454
485	A0A8H6BX17	0.10946362	1.48268809
486	A0A8H6BTW8	0.76435899	-0.04000434
487	A0A8H6C1S3	0.08569786	-0.59524504
488	A0A8H6BXN9	0.24593306	-0.35553062
489	A0A8H6BX23	0.60291383	-0.05093866
490	A0A8H6F1E4;A0A8H6F2J3	0.00567613	1.0506897
491	REVERSE1968	0.00175124	0.83597586
492	A0A8H6F1I5	0.00377773	1.46562294
493	A0A8H6F469;A0A8H6C2H9	0.13116765	-0.77269478
494	A0A8H6F3Z2;A0A8H6F5V2	0.00292647	0.39884206
495	A0A8H6F0C7	0.1816588	0.29141865
496	A0A8H6BTU2	0.80587342	-0.30540587
497	A0A8H6F5E2	0.74049705	0.05323188
498	A0A8H6C2N8	0.52410675	0.14600893
499	A0A8H6C526	0.46264586	0.13674895
500	A0A8H6F1N2;A0A8H6F289	0.80609383	-0.0634079
501	A0A8H6F102	0.08846367	0.4320996
502	A0A8H6BZR8	0.00543217	0.34132059
503	A0A8H6BVX8;A0A8H6BZ49;A0A8H6BV00	0.07268394	-0.52601451
504	A0A8H6C0C7	0.31232901	-0.21655161
505	A0A8H6BUA6;A0A8H6BS38	0.44567565	-0.079073

506	A0A8H6F188	0.10415606	-0.37223098
507	A0A8H6F2L9;G1UAT4	0.23058287	-0.39809141
508	A0A8H6BVB2	0.06920688	-1.46910484
509	A0A8H6F070	0.00242462	-0.75964791
510	A0A8H6BUW8	0.00948518	0.89442515
511	A0A8H6BSC5	0.06253652	0.32665051
512	A0A8H6BRY0	0.49935368	0.24896795
513	A0A8H6BUX9	0.04277837	1.05258178
514	A0A8H6BXZ5	0.00085905	-0.58874803
515	A0A8H6C2S4	0.5581754	0.18232693
516	A0A8H6F777	0.48436335	-0.09597462
517	A0A8H6F3A4	0.74839091	0.06980575
518	A0A8H6C0A5	0.00209421	0.89739895
519	A0A8H6BT58;A0A8H6BV62	0.78778438	-0.24253178
520	A0A8H6C051	0.08983178	0.39940136
521	A0A8H6C3S1	0.40182114	0.18127029
522	A0A8H6BZC8;A0A8H6F080	0.98077982	-0.00706031
523	A0A8H6C0F6	0.59897148	0.59132877
524	A0A8H6BVD7	0.00191559	-1.03374336
525	A0A8H6C273	0.00092915	-0.88565196
526	A0A8H6F075	0.12895054	0.28148868
527	A0A8H6C180	0.43591257	-0.10908511
528	A0A8H6BUP2	0.74867126	-0.03584493
529	A0A8H6BS90	0.23399564	0.25851052
530	A0A8H6F1J0	0.8560248	0.02401296
531	A0A8H6C0Q5	0.03503154	0.45981872
532	A0A8H6F366;A0A8H6BVM0	0.35408034	-0.13553656
533	A0A8H6C182	0.00484892	-0.76851219
534	A0A8H6F226	0.04825289	0.40912868
535	A0A8H6F581	0.42165194	-0.20689797
536	A0A8H6F210	0.63209821	-0.20621379
537	A0A8H6F1G7	0.07425217	0.22734003
538	A0A8H6C4A0	0.01171432	0.31534875
539	A0A8H6BZ27;A0A8H6BSH0	0.52349855	-0.09056876
540	A0A8H6BSU1	0.435223	0.10577569
541	A0A8H6BTG9	0.39287392	0.22427193
542	A0A8H6BZ19	0.35098658	0.1236892
543	A0A8H6F5C8;A0A8H6F5Z9	0.00368584	-0.61881713
544	A0A8H6BR37	0.03477616	0.57797408
545	A0A8H6BZY5	0.92375593	0.0193952
546	A0A8H6F6Y0	0.00243098	-0.7690724
547	A0A8H6F020	0.00181884	-0.71458812
548	A0A8H6BSY8	0.2029117	-0.24628319
549	A0A8H6C1Z5	0.15245851	0.42132087
550	A0A8H6F3V1	0.01696322	-0.39844336
551	A0A8H6F4B4	0.67597194	-0.08286226
552	A0A8H6F3S1	0.04134293	0.96092528

553	A0A8H6F138	0.44507655	0.29807044
554	A0A8H6BY07	0.44959411	-0.09880645
555	A0A8H6BYS4	9.08E-05	1.3970245
556	A0A8H6BR76;A0A8H6BUL8	0.23982461	0.22053584
557	A0A8H6F5W2	0.34732452	-0.4279526
558	A0A8H6F0V8	0.01972425	0.50618541
559	A0A8H6F273	2.43E-05	1.82606869
560	A0A8H6BZ98	0.23152435	-0.25920445
561	A0A8H6F5R8	0.34674703	0.1319587
562	A0A8H6C056	0.05773432	1.06413059
563	A0A8H6C4L0	0.01458442	0.79658157
564	A0A8H6F5N1	0.0008048	1.24273767
565	A0A8H6C673	0.13543128	0.14045737
566	A0A8H6BZH3	0.96369575	-0.01660119
567	A0A8H6BUX2;A0A8H6BSY6	1.21E-05	1.40021573
568	Q2TU56	0.29635712	0.60544197
569	A0A8H6F6C5	0.46636519	-0.10984383
570	A0A8H6F778	0.14200131	-0.34199847
571	A0A8H6BXZ7	0.33556703	0.2181485
572	A0A8H6C566	0.00130304	0.88815167
573	A0A8H6F372;Q9P8U8	0.03441884	0.45411682
574	A0A8H6C0E1;A0A8H6C0U4	0.10655947	0.42275564
575	A0A8H6BZG5	0.38797475	0.28578178
576	A0A8H6F2K8	0.62654855	-0.09687081
577	A0A8H6F317	0.37219115	0.32183699
578	A0A8H6F4R1	0.00060658	2.32030881
579	A0A8H6BXD1	0.57875618	0.07451045
580	A0A8H6BTQ6;A0A8H6F669	0.04935819	-0.42582155
581	A0A8H6BUR2	0.44918828	0.55764252
582	G1UAR3	0.39725106	-0.25023589
583	A0A8H6C024	0.34672267	-0.10428042
584	A0A8H6F2S4	0.01662868	-0.4972126
585	A0A8H6F2L7	0.00162248	0.85874042
586	A0A8H6BT21	0.04957445	0.3656687
587	A0A8H6BX16	0.00034461	1.41469501
588	A0A8H6BTE2;A0A8H6BUL2;A0A8H6BVX3	0.97867152	0.06657855
589	A0A8H6F5U2	0.111911	-0.39695843
590	A0A8H6BY02;A0A8H6F6Q5	0.21285084	-0.24333005
591	A0A8H6C403	0.08646068	0.62438031
592	A0A8H6F649	0.55457015	0.1901285
593	A0A8H6F404	0.00322625	0.65137391
594	A0A8H6C5W3	0.00221446	-0.92805873
595	A0A8H6C505;A0A8H6F5L6	0.05355678	-0.14407879
596	A0A8H6F1L5	0.94863693	-0.02323067
597	A0A8H6C1N6;A0A8H6F0J7	0.03592633	0.2931155
598	A0A8H6EZS7	0.04286191	0.43371392

599	A0A8H6C043	0.00380019	-0.96576996
600	A0A8H6BVL8	0.56858719	0.14191004
601	A0A8H6BZP2	0.25735981	-0.18527801
602	A0A8H6F3S0	0.00423295	0.80202177
603	A0A8H6BTG1;A0A8H6F225;A0A8H6F338	0.00521312	-0.99506831
604	A0A8H6C3Q1	0.19123042	-0.28825856
605	A0A8H6BVC1	0.08274836	0.52793349
606	A0A8H6F257	0.00512092	-0.52689603
607	A0A8H6C063	0.26691098	0.12061685
608	A0A8H6BXM2	0.93216345	0.01341568
609	A0A8H6C3Y7	0.64820168	0.16165495
610	A0A8H6F2H7	0.01084314	0.34711909
611	A0A8H6BT06	0.05596516	0.58192339
612	A0A8H6BYJ8	0.80333077	-0.04254024
613	A0A8H6C598	0.25009697	-0.39058923
614	A0A8H6F309	0.0466135	0.41001266
615	A0A8H6BUR3	0.87347636	0.04127989
616	A0A8H6BTI5	2.91E-05	1.36257251
617	A0A8H6BUU2;A0A8H6C4C2	0.00060411	-1.06270003
618	G1UAJ1	0.74142208	-0.01217259
619	A0A8H6BWG2	0.14229788	-0.22591656
620	A0A8H6F520	0.00194606	0.91037373
621	A0A8H6F398	0.04027574	1.05830186
622	A0A8H6F0Z1	0.12762144	-0.26836767
623	G1UAV0	0.33268533	0.1326751
624	A0A8H6BT85	0.35018875	0.2391485
625	A0A8H6C0L4	0.81894161	-0.02061418
626	A0A8H6BY55;A0A8H6F483	0.63910732	0.11203219
627	A0A8H6BWE9;A0A8H6BX58;A0A8H6BVV2	0.01681625	0.61705706
628	A0A8H6BRU9	0.04701156	0.38036883
629	A0A8H6BU20	0.00149095	1.2691509
630	A0A8H6C2D6	0.35815952	0.14224079
631	A0A8H6BTG2;A0A8H6BVH8	0.9514275	0.01393539
632	G1UAX7	0.17781807	0.34153774
633	A0A8H6F718	0.09278904	-0.46348129
634	A0A8H6C4Z9	0.77561022	-0.16562782
635	A0A8H6C308	0.89228427	-0.01577822
636	A0A8H6BTP2	0.27537036	-0.29280408
637	A0A8H6C155	0.11369026	-0.59275322
638	A0A8H6BSX1;A0A8H6BUV0	0.38453555	-0.12368469
639	A0A8H6F090	0.16113722	-0.28932325
640	A0A8H6F525	2.20E-05	-2.08477309
641	A0A8H6BTZ4;A0A8H6BVA1	0.16000632	-0.27401994
642	A0A8H6F3P9	0.00291796	0.75561088
643	A0A8H6C3A7	0.22025409	0.32151414

644	A0A8H6BRU2	0.19903712	-0.30552596
645	A0A8H6C3T5;A0A8H6C1B7	0.07581257	-1.55349649
646	A0A8H6C3W4	0.00477806	-0.67444321
647	A0A8H6BXA0;A0A8H6C4V1	0.00811249	-0.43916481
648	A0A8H6F1H8	0.01260034	-0.79902679
649	A0A8H6F478	0.94796072	0.05969685
650	A0A8H6BXF1	0.36467078	0.14463244
651	REVERSE6247	0.67720468	-0.04458602
652	A0A8H6BWP4	0.00491276	0.43069791
653	A0A8H6C005;A0A8H6C0M8	0.77709051	-0.11536257
654	A0A8H6BSP3	0.14754996	0.26421833
655	A0A8H6F2Z4	0.00231538	0.75718885
656	A0A8H6C640	0.85718819	-0.04525912
657	A0A8H6C1R1	0.49247247	-0.26277948
658	A0A8H6BZM1	0.8870577	-0.02251448
659	A0A8H6F1L3	0.25097316	0.20638787
660	A0A8H6F1Q5	0.13417518	0.55830349
661	A0A8H6F092	0.06581046	-0.20283741
662	A0A8H6C5T6	0.42608819	-0.17470758
663	G1UAD2	0.00683811	0.52900207
664	A0A8H6F2Z9	0.8524833	0.10103841
665	A0A8H6BY29	0.86176555	0.04068847
666	A0A8H6C1I1	0.01456115	0.38933293
667	A0A8H6BW37	0.00728168	0.22301307
668	A0A8H6C3D3;A0A8H6BYB5;A0A8H6BYB9;A0A8H6C1X2;A0A8H6C246;A0A8H6C2A9;A0A8H6C4P9;A0A8H6C5F1;A0A8H6C5H0;A0A8H6F021;A0A8H6F120;A0A8H6F1V8;A0A8H6F214;A0A8H6F2I5;A0A8H6F549;A0A8H6F607;A0A8H6C3L1;A0A8H6BVQ0;A0A8H6F1J4;A0A8H6F7D7;A0A8H6BU92	0.58503196	-0.2218393
669	A0A8H6C071	0.00987175	0.96669439
670	A0A8H6BZX8	0.07450492	0.30965199
671	A0A8H6C234	0.74483298	0.0919186
672	A0A8H6F1P6	0.00442806	0.24446833
673	A0A8H6C4T9	0.01514575	1.53380827
674	A0A8H6BVP0	0.18119012	0.11104026
675	A0A8H6BTK9	0.74713531	-0.04648351
676	A0A8H6BZY8	0.46947997	0.3799606
677	A0A8H6C5E1	0.33779323	0.41772463
678	A0A8H6C164	6.68E-05	-0.46253109
679	A0A8H6BSQ6	0.00024845	0.79857956
680	A0A8H6F5H5	0.16087796	0.88655445
681	A0A8H6BRF0	0.57539985	0.20663593
682	A0A8H6C4P0	0.93555741	-0.01316218
683	A0A8H6F473	0.19711536	-0.29748068

684	A0A8H6BZJ7	0.00232172	1.41516808
685	G1UAU9	0.73723389	-0.0470959
686	A0A8H6BQX0	0.00361009	-0.5856602
687	A0A8H6BSW4;A0A8H6F566	0.49362314	0.67162016
688	A0A8H6F5A0	0.86867604	-0.00859495
689	A0A8H6F222	0.39860529	0.23962279
690	A0A8H6F149	0.00581347	-0.95587036
691	A0A8H6BYL3	0.38551569	-0.37707698
692	A0A8H6C523	0.73468792	0.06140123
693	A0A8H6C2B1	0.63538393	-0.13070487
694	A0A8H6F4Y6	0.01782707	0.76093334
695	A0A8H6BYP6	0.43781505	0.18044349
696	A0A8H6C1Q8	0.59653032	0.09653201
697	A0A8H6BWL9	0.05365901	0.19559857
698	A0A8H6C1H1	0.26392246	-0.32045266
699	A0A8H6F6D5	0.02718589	1.1809172
700	A0A8H6F1B4	0.03048152	0.55102957
701	A0A8H6BZY7	0.08478237	0.61783888
702	A0A8H6F0P3	0.0025741	0.57526949
703	REVERSE4624	0.79689884	0.06117951
704	A0A8H6F0P4	0.0275883	0.48377107
705	A0A8H6F3P4;A0A8H6C6A4	0.00085977	-1.31859064
706	A0A8H6BVS9	0.65699101	0.31320901
707	A0A8H6F4E6	0.19566308	0.40178356
708	A0A8H6C3Z5	0.0672867	0.37105231
709	A0A8H6BSF4	0.08918921	0.48824417
710	A0A8H6BVX7	0.52098468	0.26192945
711	A0A8H6BTR6	0.14220001	-0.38666387
712	A0A8H6C257	0.60856589	0.01991796
713	A0A8H6BTQ4	0.03122631	-0.2577179
714	A0A8H6F496	0.01145114	0.44263497
715	A0A8H6BVL5	0.11007179	-0.85878656
716	A0A8H6C2S1	0.41293747	0.14229742
717	A0A8H6C4N9	0.30056027	-0.45888126
718	A0A8H6BTV7	0.1102863	-0.23296853
719	REVERSE2383	0.08689525	-0.45715284
720	A0A8H6C4L6	0.00134026	0.69513453
721	A0A8H6C3J4	0.39583341	-0.14449827
722	A0A8H6C0I9	0.15714453	-0.38565147
723	A0A8H6BXL2	0.23019365	0.61727635
724	A0A8H6C188;A0A8H6C3T8	0.45336595	-0.30328583
725	A0A8H6BYL7	0.37286402	-0.28614854
726	A0A8H6C3M4	0.51989738	-0.19140053
727	A0A8H6F2M6	0.19121286	-0.28218167
728	A0A8H6BU61;A0A8H6BX00	0.03243809	1.02654831
729	A0A8H6F3Z1	0.1044959	-0.16486916
730	A0A8H6BWG9;A0A8H6BVX4	0.02783938	0.44174418

731	A0A8H6BTB4	0.53584535	0.15834153
732	A0A8H6C339;A0A8H6F624	0.07025965	0.97995241
733	A0A8H6BR04	0.23615908	-0.59805541
734	A0A8H6F433	0.44009687	-0.25577464
735	A0A8H6EZU9	0.75183213	0.07269381
736	A0A8H6BV69	0.01118637	0.94212886
737	A0A8H6BSL9;A0A8H6BWU3	0.41149248	-0.11622278
738	A0A8H6C105	0.05721518	-0.38583217
739	A0A8H6F6S2	0.14427839	0.10630584
740	A0A8H6F6E7	0.89468167	-0.0967176
741	A0A8H6C1J6	0.00771735	0.94137236
742	A0A8H6EZS5	0.79270982	-0.06605099
743	A0A8H6BWD8	0.77123454	-0.0444376
744	A0A8H6BY03	0.05702181	0.67671012
745	A0A8H6BZL6	0.00754663	0.74590455
746	A0A8H6BX45	0.33369028	0.1775423
747	A0A8H6BRF4	0.37812063	-0.23927539
748	A0A8H6BRX6	0.30946021	0.10646609
749	A0A8H6BZI1	0.00052695	-1.08907453
750	A0A8H6F6H9	0.07467727	0.44159254
751	A0A8H6BZ11;A0A8H6F410	0.0355647	0.66525506
752	A0A8H6BXG8	0.01305011	1.00970177
753	A0A8H6BS19	0.48333949	0.15169108
754	A0A8H6BZQ9	0.13776049	0.36309427
755	A0A8H6BX15;A0A8H6BVR5	0.67674482	-0.07816978
756	A0A8H6BY04	0.0003836	-1.38961608
757	A0A8H6C1M9	0.815842	-0.10407634
758	A0A8H6C4D3	0.08037269	0.3193633
759	A0A8H6BVP3	0.06843147	0.46588156
760	A0A8H6F5Z8	0.00845282	0.58031276
761	A0A8H6BWQ6	0.03397643	0.4864992
762	A0A8H6F0Q4	0.92592804	0.06332463
763	A0A8H6C551	0.06197646	0.58865912
764	A0A8H6F3W0	0.16089954	0.39503202
765	A0A8H6C375	0.35293849	-0.31169223
766	A0A8H6C0I8	0.051846	-0.49386187
767	A0A8H6BUS3	0.2003481	-0.41090448
768	A0A8H6F4N6	0.00889473	1.01979341
769	A0A8H6BR20	0.14738594	-0.25443832
770	A0A8H6F2V4	0.05967093	0.52303523
771	A0A8H6F2Q0	0.44416698	0.24350157
772	A0A8H6BTF2	0.00485448	0.68342575
773	A0A8H6C1U3	0.10468592	-0.45875799
774	A0A8H6BWL4	0.00014327	1.30309173
775	A0A8H6BWW4	0.27547949	0.44812515
776	A0A8H6F4F0;A0A8H6C2Z4	0.2811348	0.31969703
777	A0A8H6BVB3	0.26511756	-0.29751164

778	Q9UUZ0	0.83197105	-0.05438993
779	A0A8H6F5F5	0.06451939	0.58409895
780	A0A8H6BSH2	0.04634065	0.33146723
781	A0A8H6BSJ4;A0A8H6F1G6	0.00961363	-0.46120905
782	A0A8H6F0X1	0.14379487	0.18767748
783	A0A8H6BYX7;A0A8H6F043	0.74876053	0.25236953
784	A0A8H6BZU6	0.10599207	0.28893277
785	A0A8H6BXS8	0.44342746	-0.18618439
786	A0A8H6C561	0.00386857	0.81970508
787	A0A8H6F184	0.36122437	0.45633313
788	A0A8H6F4J9	0.47504102	-0.14343175
789	A0A8H6C4S7	0.03886468	0.33960426
790	A0A8H6F6K3	0.14327703	-0.37854245
791	A0A8H6BSB3	0.93072607	0.00494812
792	A0A8H6BTH9	0.1036213	-0.67606042
793	A0A8H6BUF3	0.01420736	-1.32588072
794	A0A8H6F235	0.22802142	-0.92372027
795	REVERSE6168	0.01673762	0.70077438
796	A0A8H6BU19	0.10776736	0.14750791
797	A0A8H6F1A0	0.73666775	0.15217816
798	A0A8H6BVI9	0.01163236	-0.61106831
799	A0A8H6C424;A0A8H6C556	0.01771961	-0.63466303
800	A0A8H6BZA0	0.30362928	-0.39640897
801	A0A8H6BWD1	0.07696745	0.30198643
802	A0A8H6C251	0.03130541	1.00845263
803	A0A8H6C348	0.13531758	0.34194178
804	A0A8H6F381	0.09542178	0.19681149
805	A0A8H6C2P2	0.22106302	0.26324464
806	A0A8H6F3D9	0.07520649	-0.47028342
807	A0A8H6C2V4	0.80744246	-0.10269118
808	A0A8H6BTY5;A0A8H6BVT7	0.89746944	0.01629139
809	A0A8H6C5Y4	0.24582434	0.2630048
810	A0A8H6BTI6	0.03215524	-0.36981805
811	A0A8H6F6I5	0.03161101	0.62785855
812	A0A8H6BVU3	0.53417317	-0.2995196
813	A0A8H6BYN2	0.8812137	-0.06417607
814	A0A8H6BYF0	0.13989767	0.30499338
815	A0A8H6F1U5	0.72973686	0.03792913
816	A0A8H6BTB5	0.58760315	-0.08803141
817	A0A8H6F575	0.23149771	-0.3524364
818	A0A8H6BWM9	0.96408507	-0.04398785
819	A0A8H6F189	0.56760015	0.16962474
820	A0A8H6F4D7	0.46859282	0.28330109
821	A0A8H6F1E7	0.5566665	0.35403529
822	A0A8H6C4G2;A0A8H6C1N1	0.36204043	0.16259522
823	A0A8H6C146;A0A8H6BWI5	0.25028234	-1.03686241
824	A0A8H6C516;A0A8H6C2L0	0.19103936	-0.65534658

825	A0A8H6F045	0.02197987	-1.33774282
826	A0A8H6F4E0	0.39606164	-0.30581752
827	A0A8H6C1Q1	0.10982613	0.23306579
828	A0A8H6C455	0.25809569	0.22998661
829	A0A8H6BW03	0.4569869	-0.1860566
830	A0A8H6C3H0	0.29967817	0.19002565
831	A0A8H6BX78	0.65465807	-0.09769547
832	A0A8H6F041	0.62551911	0.21970408
833	A0A8H6C070	0.0301622	0.42542172
834	REVERSE2876	0.09993528	-0.26439095
835	A0A8H6C4I7;A0A8H6C4V8	0.39198198	-0.18600488
836	A0A8H6F377	9.61E-05	2.50794924
837	A0A8H6F324	0.02825715	-1.3068873
838	A0A8H6BVI0	0.95883433	0.0312515
839	A0A8H6F709	0.20173931	-0.26220461
840	A0A8H6F4G3	0.00103032	-0.72068762
841	A0A8H6C299	0.04174474	-2.03318163
842	A0A8H6BYK3;A0A8H6F5L7	0.20737467	0.3264256
843	A0A8H6BZ86	0.55721075	-0.44833955
844	REVERSE49	0.01182445	0.52881361
845	A0A8H6BS98;A0A8H6BT89;A0A8H6BUJ4	0.73970291	0.08414023
846	A0A8H6C593	0.27386183	-0.35556855
847	REVERSE759	0.13670553	0.68412731
848	A0A8H6BTN3	0.53171265	0.20717119
849	A0A8H6C124;A0A8H6F4T8	0.4930744	0.35046432
850	A0A8H6F0B1;A0A8H6F0R0	0.15035453	1.12128014
851	A0A8H6C2U9	0.95516078	-0.0079457
852	A0A8H6C4N6;A0A8H6BUG8;A0A8H6BRL7	0.00037513	-1.91127764
853	A0A8H6C5W7	0.51526302	0.27667167
854	A0A8H6C0V7;A0A8H6F1V6;A0A8H6F1A8	0.02424366	1.48787831
855	A0A8H6F0F3;A0A8H6F2D2	0.5134515	-0.15605994
856	A0A8H6F0E9	0.28470481	0.48150649
857	A0A8H6C2K1	0.30780936	0.36390007
858	A0A8H6BYI4;A0A8H6BZF1	0.02340745	-1.14508336
859	A0A8H6BYI8	0.44909241	0.23623817
860	A0A8H6C0U0	0.04050186	1.12085773
861	A0A8H6C470	0.03902792	-1.37768061
862	A0A8H6BSF9;A0A8H6BY87	0.30436327	0.14332655
863	A0A8H6F4H0	0.31647773	0.15712528
864	A0A8H6BS91	8.27E-05	0.71007736
865	A0A8H6BW12	0.01025798	-0.62652174
866	A0A8H6C075	0.01132826	0.86655248
867	A0A8H6BVR0	0.52205258	0.14513203
868	G1U9Z1	0.72962344	-0.07535784

869	A0A8H6BZJ6	0.16720711	0.38433459
870	A0A8H6F456	0.44077517	2.01919517
871	A0A8H6EZU7	0.04203807	1.04830006
872	A0A8H6C5Q0	0.13267199	0.87911388
873	A0A8H6F645	0.40658266	0.10037325
874	A0A8H6BWC0	0.17378055	0.69967392
875	A0A8H6F6B2	0.05774586	0.73872544
876	A0A8H6C1N0	0.45134444	-0.13251404
877	A0A8H6BWZ2;A0A8H6F3A1;A0A8H6BVT6	0.84883447	-0.02405843
878	A0A8H6C0Q0	0.16149401	-0.4757205
879	REVERSE76	0.99347479	0.26645851
880	A0A8H6C404	0.3390575	-0.80168091
881	A0A8H6BVM4	0.00011806	-1.34273374
882	A0A8H6BS11	0.00209201	-0.99062604
883	A0A8H6BVE4	0.02403659	-0.95747531
884	A0A8H6F0M8	0.00236598	-1.38272697
885	A0A8H6BVL9	0.21638575	0.43195139
886	A0A8H6BW70	0.17710841	-0.25530329
887	A0A8H6F4L8	0.1312861	-0.44520122
888	A0A8H6F5X2	0.5418326	-0.45541501
889	A0A8H6F3S7	0.19236027	0.20022459
890	A0A8H6F3G1	0.0296744	1.19079042
891	A0A8H6BUJ5;A0A8H6C5L3	0.18112998	0.56642863
892	REVERSE2660	0.36493052	-0.2498973
893	A0A8H6C0M4	0.71849474	-0.05447812
894	A0A8H6BXX7;A0A8H6F237	0.1178151	-0.77314208
895	A0A8H6BWX2;A0A8H6C3T7	0.05440486	-1.26988174
896	A0A8H6C0C9	0.86448023	0.12236128
897	A0A8H6C1Z0	0.35458894	-0.38103203
898	A0A8H6BRX0;A0A8H6C1G6	0.0060915	0.98996
899	A0A8H6BXH1	0.00063727	-0.85741142
900	A0A8H6C5A5	0.19714903	0.39014416
901	A0A8H6BUC2	0.32684359	-0.35228914
902	A0A8H6C204	0.09257304	-0.61373904
903	A0A8H6BZT5	0.00023388	-2.40153452
904	A0A8H6F775	0.24018327	0.60015289
905	A0A8H6BQX5;A0A8H6BTG4	0.6900974	-0.10656191
906	A0A8H6BY51	0.03637844	-0.45942538
907	A0A8H6F262	0.00900624	0.8999165
908	REVERSE1244	0.90819016	2.11420756
909	A0A8H6C0L0	0.00434468	-0.66403551
910	A0A8H6BUY8	0.71262486	-0.12080216
911	A0A8H6F639	0.25512336	0.28354975
912	A0A8H6C0P7	0.23430681	-0.58301175
913	A0A8H6BTZ5	0.43594783	0.1155656
914	A0A8H6F196	0.01367014	1.17560781

915	A0A8H6F4Q7	0.38730295	0.45203373
916	A0A8H6BYQ3	0.74117527	0.15164776
917	A0A8H6C587;A0A8H6C595	0.02367768	-1.25986693
918	A0A8H6BUB3	0.73635617	0.25248866
919	A0A8H6F5A2	0.05022358	0.8961231
920	A0A8H6C5G7	0.72529339	0.24481762
921	A0A8H6F371	0.78104232	0.11082834
922	A0A8H6F3K0;A0A8H6F475	0.43310102	-0.13989746
923	A0A8H6C2J0	0.69097976	-0.11713499
924	A0A8H6F3M0	0.42390724	-0.41053642
925	A0A8H6C1K8	0.32011465	-0.66976281
926	A0A8H6C0K5	0.69858691	-0.08937513
927	A0A8H6BYQ6	0.95105017	0.14867768
928	A0A8H6C224;A0A8H6C5F2	0.89167898	-0.06189025
929	A0A8H6C0T2;Q96UT6	0.3640106	-0.40136126
930	A0A8H6BWZ9	0.01065499	-0.40191924
931	A0A8H6C3B7;A0A8H6C646	0.43499292	-0.24555161
932	A0A8H6BRV2	0.00032149	-1.05809324
933	A0A8H6F171	0.55044249	0.60084505
934	A0A8H6F2I7	0.06299864	0.51137702
935	A0A8H6F115	0.00021216	-0.95094211
936	A0A8H6BSH7	0.05644764	-0.63148575
937	A0A8H6BUS5;A0A8H6F3F9	0.56132597	-0.10723036
938	A0A8H6BXP9	0.73248657	-0.08631959
939	A0A8H6C1G8	0.03264031	-0.4552835
940	A0A8H6BUJ3;A0A8H6BVV3	0.99659855	-0.00357386
941	A0A8H6BVJ6	0.00145328	-1.55472801
942	A0A8H6C2U7	0.89958163	-0.08145069
943	A0A8H6F6B8	0.15060626	-0.27870586
944	A0A8H6BZD6	0.01955502	0.3468628
945	A0A8H6F126	0.0002248	-1.16990666
946	A0A8H6F6T9	0.18208061	-1.11215127
947	A0A8H6F606	0.01837581	0.58139407
948	A0A8H6BX04	0.0481069	-1.22197627
949	A0A8H6BTQ1	0.0118165	0.93617078
950	A0A8H6BY99;A0A8H6C279	0.02180198	-0.65896632
951	A0A8H6F4Y8;A0A8H6F5R7	0.99251533	-0.07809614
952	A0A8H6F3W8	0.88983585	0.0967174
953	A0A8H6F198	0.20706759	0.67848125
954	A0A8H6BYI2;A0A8H6BZ22;A0A8H6BY09	0.05261236	-1.39870354
955	A0A8H6F3C7	0.93212015	0.06530794
956	A0A8H6BQX2	0.34918579	-0.30979694
957	A0A8H6BYN7	0.00664162	0.19505298
958	A0A8H6BUL9	0.18533225	0.96548178
959	A0A8H6C2S5	0.85346236	0.02772254
960	REVERSE7009	0.18646949	-0.34875496

961	A0A8H6BWM4	0.04275979	0.87198725
962	A0A8H6C138;A0A8H6C3Y4	0.89190692	0.03295191
963	A0A8H6C607	0.07858894	1.01437864
964	A0A8H6F1S2	0.9795777	0.09143045
965	REVERSE1810	0.24094429	0.23326755
966	A0A8H6F3K9	0.27574079	0.21211674
967	A0A8H6F537	0.14659271	0.90380049
968	A0A8H6BXX8	0.15702645	-0.49620069
969	A0A8H6C2J8	0.07940252	-1.81639395
970	A0A8H6BTJ8	0.02805221	1.40960955
971	A0A8H6BW71	0.10058382	1.09462102
972	A0A8H6C015	0.04857545	-1.20896913
973	A0A8H6BYX4	0.36000815	0.63846374
974	A0A8H6F619	0.05021856	-0.75817787
975	A0A8H6BWM1;A0A8H6BYY0	0.9927812	0.12433409
976	A0A8H6BWI0	0.51019331	-0.18807047
977	A0A8H6BYD9	0.55798627	0.31627299
978	A0A8H6F616	0.80332761	0.38215332
979	A0A8H6C177	0.00345736	-1.4974877
980	A0A8H6C1R4	0.0290698	1.20752267
981	A0A8H6C3F9;G1UAE7	0.02308827	-1.02193112
982	A0A8H6BZG0	0.01043543	-1.40506918
983	A0A8H6C608	0.5156949	-0.3661217
984	A0A8H6F700	0.24449036	-0.99047264
985	A0A8H6F5L2	0.06227499	-1.33433734
986	A0A8H6BWI4	0.00853897	-1.69422565
987	A0A8H6BZ64	0.02330819	-1.01673829
988	A0A8H6F4H1;A0A8H6F596	0.02455518	-2.10729989
989	A0A8H6C3K4	0.07178617	0.4379272
990	A0A8H6BV03	0.04907962	0.43219177
991	REVERSE2260	0.6234752	0.28094093
992	A0A8H6F4E1	0.86020344	0.07870499
993	A0A8H6BSH5	0.36108732	-0.63741323
994	A0A8H6BXT2	0.08492421	-1.03435807
995	A0A8H6C370;A0A8H6C5M4	0.56151624	-0.45108645
996	A0A8H6BW44;A0A8H6C241;A0A8H6F1G2	0.11968302	-0.84948988
997	A0A8H6F0Q2	0.92988443	-0.06562072
998	A0A8H6F3K7	0.17046019	0.26238937
999	A0A8H6F1H3	0.13872298	1.63062834
1000	A0A8H6C423	0.00762388	-1.10645315
1001	A0A8H6C5A2	0.0010837	-1.29405724
1002	A0A8H6C4B6	0.14514544	0.82920789
1003	REVERSE6294	0.15531496	0.92354536
1004	REVERSE4680	0.01515293	1.51054027
1005	A0A8H6C0Z7	0.16474023	-0.89621169
1006	A0A8H6BTL0	0.9845209	0.02960163

1007	A0A8H6BSI1	0.71807401	-0.08876625
1008	A0A8H6F180	0.30647607	0.86037285
1009	A0A8H6C4C3	0.24985503	0.53811396
1010	A0A8H6C2Q4	0.05691221	-1.19643845
1011	A0A8H6C2A0	0.32845875	-0.82854534
1012	A0A8H6C4B5	0.24495964	-0.95512067
1013	A0A8H6BUR0	0.94074304	-0.00768424
1014	A0A8H6C1J4	0.34912061	3.98138313
1015	A0A8H6BQV5	0.06155343	-1.40256507
1016	A0A8H6F154	0.94015172	-0.01829357
1017	A0A8H6F1P2	0.7575881	0.21980768
1018	A0A8H6C2V1	0.76892462	0.08405866
1019	A0A8H6BUW4	0.00946742	-1.58449721
1020	REVERSE1405	0.05522283	-0.84422386
1021	REVERSE4057	0.15247498	-1.62118347
1022	A0A8H6F1Q2	0.02610214	0.54722702
1023	A0A8H6F671	0.06158781	-0.34405036
1024	A0A8H6F2H6	0.28887235	0.46626072
1025	G1UA99	0.25809746	-0.47737247
1026	A0A8H6BYA8;A0A8H6C1U5	0.01047433	-0.8954627
1027	A0A8H6C1X5	0.45065797	-0.45331805
1028	A0A8H6F3J9	0.52864014	0.43292176
1029	A0A8H6BTP1	0.40991052	0.49221551
1030	A0A8H6BYH8	0.49441879	0.56059905
1031	REVERSE2976	0.00497625	2.02853671
1032	A0A8H6BRV3	0.34580145	-0.65695548
1033	A0A8H6C2E2	0.59942225	-0.33608284
1034	A0A8H6BTS0	0.03542047	0.97411515
1035	A0A8H6BVI4	0.0084423	-1.74625075
1036	A0A8H6F320	0.38835346	-0.36563271
1037	REVERSE3934	0.010157	-1.7285294
1038	REVERSE2244	0.5301167	0.33523266
1039	A0A8H6BRZ2	0.01701691	0.42441651
1040	A0A8H6F4W5	0.60058804	-0.77116533
1041	A0A8H6F3R0	0.01305729	-1.43699513
1042	A0A8H6F3T6	0.00365639	-1.78557143
1043	A0A8H6F0G1	0.30653057	0.58573673
1044	A0A8H6BT99	0.07461103	0.94780894
1045	A0A8H6BT67	0.94333062	0.13575728
1046	A0A8H6BTE0	0.72768941	0.29676173
1047	A0A8H6BYB4;A0A8H6C665	0.45140598	1.81088679
1048	A0A8H6C5J6	0.65885797	-0.27013567
1049	A0A8H6C2T8	0.46883211	1.07442936
1050	A0A8H6BVL1	0.0150769	-1.80213511
1051	A0A8H6C2Z8	0.06492184	1.14407285
1052	A0A8H6BX48	0.52274123	0.41812097
1053	A0A8H6BWL8	0.96493177	0.0382485

1054	G1UAR6	0.00160136	-2.16485407
1055	A0A8H6F4V1	0.0848243	-0.30127002
1056	A0A8H6C0C1	0.01446041	-2.54998877
1057	A0A8H6C3S6	0.01973666	-1.24857685
1058	REVERSE707	0.05711368	-1.35686121
1059	A0A8H6F5Q9	0.12242983	-0.70300393
1060	REVERSE6418	0.00168741	-1.22106538
1061	A0A8H6BYX9	0.00862159	-1.88715079
1062	A0A8H6F0D5	0.67276583	-0.98904574
1063	A0A8H6F2D3	0.09137816	-0.96528867
1064	A0A8H6BXH3	0.13088457	-2.67542687
