# Supplementary figures and images for: Cell Wall Proteome of Candida albicans Reveals Proteins Associated with Tolerance to Antibiofilm Activity of a Lippia graveolens Kunth Stem Extract
Source: Pathogens. 2026 Feb 14;15(2):216. doi: 10.3390/pathogens15020216 (PMC12942757; doi:10.3390/pathogens15020216)

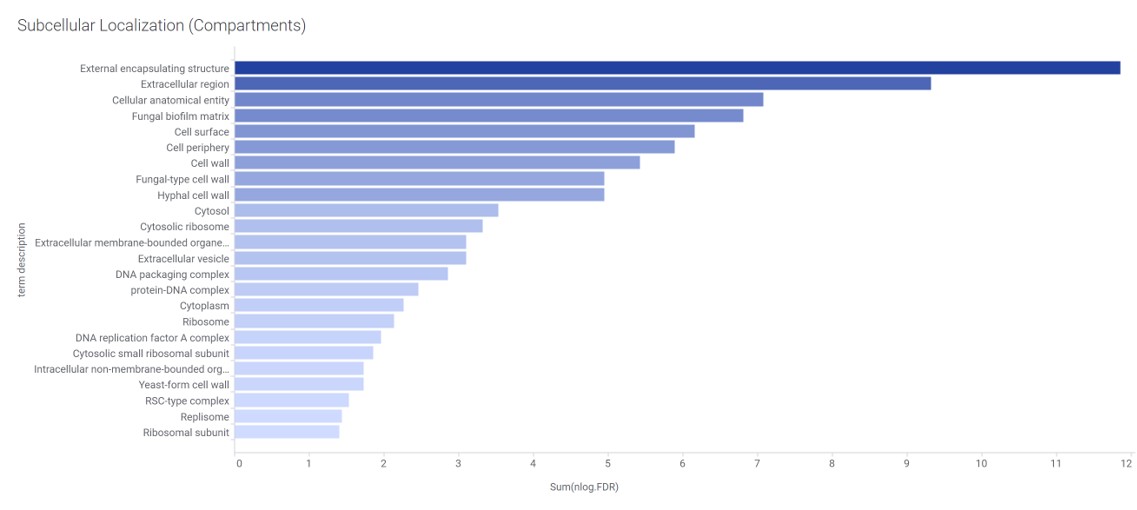

Supplement: Supplementary file 1 [file pathogens-15-00216-s001.zip › Figure S2. Subcellular localization (compartments)..jpg]
